# Soil microbial community structure and catabolic activity are significantly degenerated in successive rotations of Chinese fir plantations

**DOI:** 10.1038/s41598-017-06768-x

**Published:** 2017-07-27

**Authors:** Zeyan Wu, Jianjuan Li, Jie Zheng, Jinfu Liu, Shuying Liu, Wenxiong Lin, Chengzhen Wu

**Affiliations:** 1 Life Sciences College of Fujian Agriculture and Forestry University, Fujian, 350002 China; 2 Fujian Provincial Key Laboratory of Agroecological Processing and Safety Monitoring, School of Life Sciences, Fujian Agriculture and Forestry University, Fuzhou, 350002 China; 3 Key Laboratory of Crop Ecology and Molecular Physiology (Fujian Agriculture and Forestry University), Fujian Province University, Fuzhou, 350002 China; 4 Forestry College of Fujian Agriculture and Forestry University, Fujian, 350002 China

## Abstract

This study examined the hypotheses that soil microbial community composition and catabolic activity would significantly degenerated by consecutive monoculture in Chinese fir plantations. The phospholipid fatty acids (PLFA) and community level physiological profiles (CLPP) methods were used to assess the variations of soil microbial community among the first rotation Chinese fir plantation (FCP), the second rotation plantation (SCP) and the third rotation plantation (TCP). The total content of PLFA biomarkers was highest in FCP, followed by SCP, and TCP was the least detected. Conversely, the fungi/bacteria ratio significantly increased in the SCP and TCP soils. The average well-color development (AWCD) values significantly decreased (FCP > SCP > TCP). However, the sum of AWCD values of amino acids, carboxylic acids and phenolic compounds were higher significantly in the SCP and TCP soils than FCP soils, suggesting that the microflora feeding on acids gradually became predominant in the continuous monoculture plantation soils. Soil C/N ratio was one of the most important factors to soil microbial diversity. Both the PLFA and CLPP results illustrated the long-term pure plantation pattern exacerbated the microecological imbalance in the rhizospheric soils of Chinese fir, and markedly decreased the soil microbial community diversity and metabolic activity.

## Introduction

Chinese fir (*Cunninghamia lanceolata* (Lamb.) Hook), which covers over 12 million ha in China, is a famous coniferous timber species because of its high yield and fast-growing^1–3^. However, studies during the past few decades have reported that regeneration failure and productivity decline were observed in long-term monoculture Chinese fir plantations, referred to as consecutive monoculture problem (CMP)^4–6^. Many cultivated tree species around the world suffer from CMP, such as *Pinus elliottii*, *Picea mariana*, *Picea abies*, *Pinus halepensis* and *Eucalyptus* spp.^7–9^. Previous studies have shown that there are usually three reasons result in CMP: the soil nutrient depletion^10^, the autotoxicity of root exudates^11^ and the unbalance of soil microflora^12, 13^. Much work has been conducted to investigate the origin of CMP in Chinese fir plantations. Some researchers have found that continuous monoculture could cause depletion of nutrient elements in Chinese fir stands^14^, but CMP can’t be solved by enhancing the chemical fertilizer^15^, implying that CMP is more related to autotoxicity and soil microorganisms^16^. Although a large body of documents have focused on the autotoxicity of root exudates, much controversy still exists^17^. Moreover, the toxic substances are not directly acting on plants but restricted by rhizosphere microorganisms^18^. With the in-depth study of rhizosphere ecology, the research on CMP gradually came to focus on the rhizospheric biological processes^19^. Therefore, scientists have pointed out that plant can’t be separated from the rhizosphere soil microbiome. The deep understanding of plant-soil-microbe interactions mediated by rhizospheric biological processes has important implications for elucidating the mechanisms of CMP. However, the research on soil microorganisms in rhizosphere of Chinese fir plantations is still limited.

Soil microbes is the crucial component of forest ecosystem. The complex microbial community in soil rhizosphere is referred to as the second genome of the plant, and the cross-talk between plants and microbes is also referred to one of the key factors of CMP^20^. The interaction between CMP and soil microorganism has become an advanced research hotspot in recent years^21, 22^. For instance, Chen *et al*. found that soil bacterial communities had significant changes under continuous peanut cultivation^23^. In this study presented here, we addressed the hypotheses that soil microbial community composition and metabolic activity would significantly degenerated by consecutive monoculture in Chinese fir plantations. The objectives of our research were to answer two questions: (a) What’s the effect of consecutive monoculture on soil microbial community in Chinese fir plantations and (b) What are the key factors resulting in the variations of soil microbial community?

To describe the composition of the microbial communities in forest soils, the culture independent methods have been widely applied. Compared to other methods, phospholipids fatty acid analysis (PLFA) and community level physiological profiles (CLPP) methods are quantitative, have a relatively high throughput, and allow rapid analyses for the high number of samples needed for field-based microbial ecology investigations. Therefore, PLFA and CLPP methods were applied to assess the soil microbial community of long-term monoculture Chinese fir plantations. Our study will help to further understand ecological linkages between aboveground vegetations and underground microbes, and will therefore facilitate the establishment of scientific-based, effective management to achieve a better balance in the forest ecosystem.

## Results

### The characteristics of each experimental plots

The characteristics of each experimental plots were list in Table 1. According to Table 1, the pH values ranged from 5.28 ± 0.21 to 5.64 ± 0.33, suggesting that all the soil samples were acidic. The values of TOC, TN, AN, AK in FCP soils were significantly higher than SCP and TCP soils, indicated that continuous monoculture indeed caused depletion of nutrient elements in Chinese fir stands. Conversely, the value of C/N ratio increased with the increasing rotations in Chinese fir plantations.

**Table 1:** Characteristics of experimental plots.

Characteristics	FCP	SCP	TCP
Altitude (m)	232	275	239
Slope	30°	31°	28°
Slope aspect	ES40°	ES35°	ES40°
Average diameter at breast height (cm)	17.39	13.27	11.94
Average height (m)	13.61	10.52	9.03
Stand density (stem/ha)	930	945	945
Soil pH	5.83 ± 0.26a	5.64 ± 0.33a	5.28 ± 0.21a
Total organic carbon (TOC, g·kg^−1^)	23.54 ± 0.30a	20.09 ± 0.46c	21.37 ± 0.15b
Total nitrogen (TN, g·kg^−1^)	1.23 ± 0.01a	0.98 ± 0.01b	0.96 ± 0.01b
Available nitrogen (AN, mg·kg^−1^)	35.84 ± 0.88a	30.42 ± 0.97b	26.11 ± 1.28c
Total phosphorus (TP, g·kg^−1^)	0.45 ± 0.01a	0.40 ± 0.01a	0.33 ± 0.01b
Available phosphorus (AP, mg·kg^−1^)	3.28 ± 0.11ab	3.56 ± 0.14a	2.97 ± 0.15b
Total potassium (TK, g·kg^−1^)	18.51 ± 0.45a	17.38 ± 0.29a	15.74 ± 0.38b
Available potassium (AK, mg·kg^−1^)	140.66 ± 2.65a	125.17 ± 3.17b	123.29 ± 1.97b
C:N	19.13	20.50	22.26

### Soil microbial community composition

A total of 21 different PLFAs were identified from all soil samples (Table 2; See the Supplementary Tables S8–S10 for details). The total content of PLFA biomarkers was highest in FCP (102.18 ± 1.15 ug·g^−1^), followed by SCP (94.69 ± 0.96 ug·g^−1^), and TCP was the least detected (89.48 ± 0.64 ug·g^−1^). The cy19:0 was the dominant microorganisms in FCP (15.37 ± 0.13 ug·g^−1^), while the i16:0 was dominant microorganisms in SCP (17.54 ± 0.27 ug·g^−1^) and TCP (19.63 ± 0.22 ug·g^−1^). In FCP soil, the sum of 16:00, i17:0, i16:0 and cy19:0 account for 51.56% of the total PLFAs. In SCP soil, the sum of i17:0, i16:0, cy19:0 and 18:3ω6c(6,9,12) account for 53.76% while in TCP soil, the sum of 16:00, i16:0, cy19:0 and 18:3ω6c(6,9,12) account for 59.30%. Therefore, the top ranked PLFAs in all soil samples were 16:00, i17:0, i16:0, cy19:0 and 18:3ω6c(6,9,12).

**Table 2:** Types and contents of PLFAs in three rotations plantations (ug·g^−1^).

No.	Biomarkers	Microbial group	Stand ages		
			FCP	SCP	TCP
1	16:00	Gram(−) bacteria	9.96 ± 0.20a	8.54 ± 0.09b	7.23 ± 0.14c
2	i17:0	Gram(+) bacteria	12.47 ± 0.17a	9.08 ± 0.19b	5.49 ± 0.07c
3	cy17:0	Gram(−) bacteria	3.98 ± 0.08a	3.10 ± 0.05b	2.11 ± 0.05c
4	10Me16:0	Actinomycete	4.29 ± 0.11a	4.45 ± 0.04a	4.51 ± 0.07a
5	16:1ω7c	Gram(−) bacteria	1.25 ± 0.04a	0.88 ± 0.02b	0.52 ± 0.01c
6	i18:0	Gram(+) bacteria	5.66 ± 0.24a	4.78 ± 0.09b	3.54 ± 0.15c
7	i14:0	Gram(+) bacteria	1.59 ± 0.04a	1.28 ± 0.03b	1.01 ± 0.01c
8	i16:0	Gram(+) bacteria	14.88 ± 0.25c	17.54 ± 0.27b	19.63 ± 0.22a
9	cy19:0	Gram(−) bacteria	15.37 ± 0.13a	13.22 ± 0.11b	13.57 ± 0.14b
10	20:4ω6c(6,9,12,15)	Protozoon	0.45 ± 0.01b	0.58 ± 0.03a	0.62 ± 0.01a
11	18:3ω3	Fungi	1.67 ± 0.02c	1.94 ± 0.02b	2.53 ± 0.02a
12	a16:0	Gram(+) bacteria	1.65 ± 0.08	—	—
13	a17:0	Gram(+) bacteria	3.42 ± 0.10a	2.65 ± 0.06b	2.51 ± 0.10a
14	i15:0	Gram(+) bacteria	4.96 ± 0.22a	4.15 ± 0.11b	3.33 ± 0.08c
15	15:0	Gram(+) bacteria	6.05 ± 0.07a	4.41 ± 0.05b	3.62 ± 0.06c
16	18:3ω6c(6,9,12)	Fungi	8.19 ± 0.11c	11.07 ± 0.14b	12.63 ± 0.12a
17	16:1ω9c	Gram(+) bacteria	0.85 ± 0.02a	0.94 ± 0.03a	—
18	9Me18:0	Actinomycete	2.53 ± 0.06b	2.58 ± 0.03b	3.42 ± 0.05a
19	18:3w3	Fungi	1.06 ± 0.01b	1.93 ± 0.02a	2.09 ± 0.02a
20	16:1ω9t	Gram(−) bacteria	0.48 ± 0.01a	0.52 ± 0.01a	0.49 ± 0.01a
21	a15:0	Gram(+) bacteria	1.42 ± 0.02a	1.05 ± 0.01b	0.63 ± 0.01c
Total content of different PLFA biomarkers			102.18 ± 1.15a	94.69 ± 0.96b	89.48 ± 0.64c

The statistics of major microorganism groups were listed in Table 3. In all soil samples, the bacterial PLFAs was higher than fungi and actinomycetes. Gram(+) bacteria and Gram(−) bacteria were lowest in TCP, with values in FCP soil equaling 86.49% and 77.06% respectively. However, the amount of fungal PLFAs was lowest in FCP and highest in TCP. Accordingly, the fungi/bacteria ratio (F/B) also lowest in FCP and highest in TCP. Actinomycetes PLFAs did not change significantly in three rotation plantations.

**Table 3:** The statistics of major microorganism groups (ug·g^−1^).

Major microorganism groups	Different generations		
	FCP	SCP	TCP
Bacteria	83.99 ± 1.47a	75.74 ± 1.32b	69.72 ± 0.86c
Gram(+)	52.95 ± 0.50a	49.48 ± 0.37b	45.80 ± 0.21c
Gram(−)	31.04 ± 0.36a	26.26 ± 0.19b	23.92 ± 0.14c
Fungi	10.92 ± 0.11c	14.67 ± 0.18b	16.39 ± 0.23a
Actinomycete	6.82 ± 0.09b	7.03 ± 0.08b	7.93 ± 0.13a
Gram(+)/Gram(−) (%)	170.59	188.42	191.47
Fungi/Bacteria (%)	13.00	19.37	23.51

**Figure 1:**
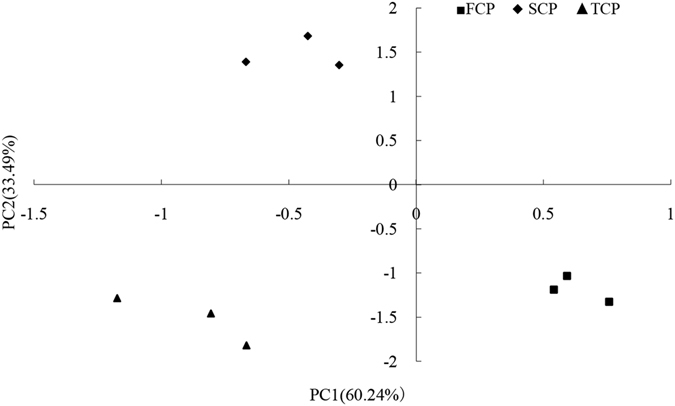
Principal components analysis (PCA) of three rotations plantations.

The changes of microbial communities in different soil samples were analyzed by principal component analysis (PCA). The PCA score plot revealed that the structures of soil microbial community in the FCP, SCP and TCP sites were clearly different from each other, with SCP and TCP on the left side of the axis, and FCP on the right side, which described 60.24% and 33.49% of the total variance, respectively (Fig. 1).

### Soil microbial catabolic activity

**Figure 2:**
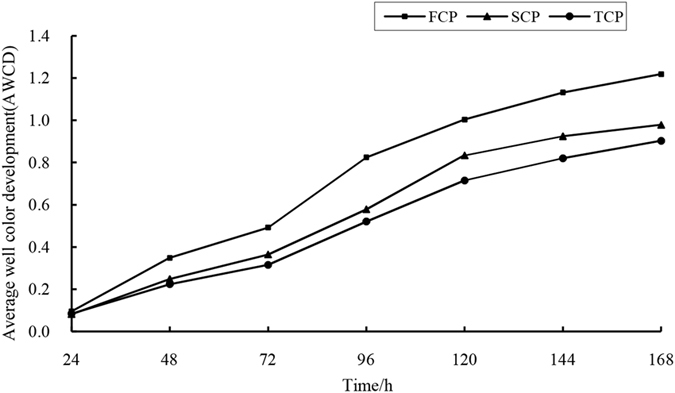
Changes of AWCD of soil microbe community with incubation time in different generations.

The AWCD values, which characterizes the catabolic activity of soil microbial community, increased with the incubation time in all soil samples, and showed a typical sigmoid course curve across the 168 h (Fig. 2; See the Supplementary Tables S1–S7 for details). On the whole, the AWCD values of all soil samples were low within 24 h, and the values increased over the time after 24 h. The AWCD values’ growth rate were highest during in 72–96 h, and the values changed gradually slow after 96 h. However, the utilization rate of FCP soil was much higher than SCP and TCP soils, indicated that the soil microbial catabolic activity decreased with the increasing planting rotations (FCP > SCP > TCP).

**Figure 3:**
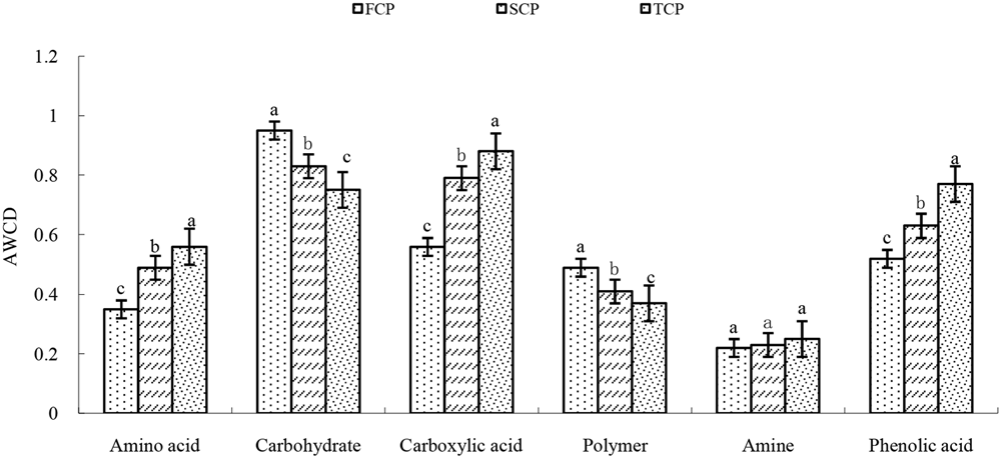
Mean catabolic activity per well for each substrate group in different generation soils.

**Figure 4:**
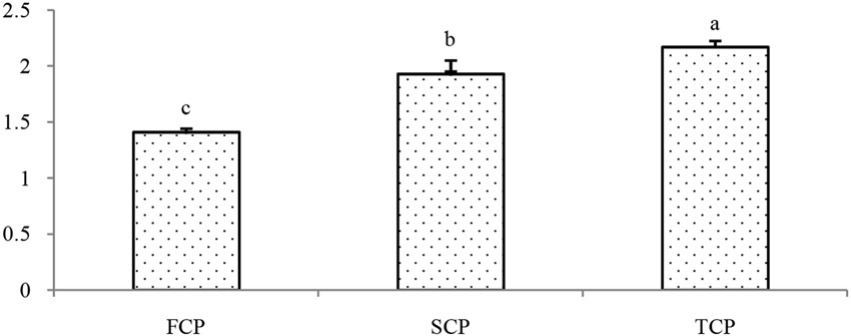
Sum of AWCD values of amino acids, carboxylic acids and phenolic compounds at 96 h.

The total 31 single carbon substrates were divided into 6 categories: amino acids, carbohydrates, carboxylic acids, polymers, amines and phenolic acids. Generally speaking, 96 h is the time of most active microbial community reach the asymptote of the color change during culture time. Except for carbohydrates and polymers, the 96 h AWCD data of the other four substrate groups (amino acid, carboxylic acids, amines and phenolic acids) were all highest in the TCP soil and lowest in the FCP soil (Fig. 3). The microbial communities from the continuous monoculture of Chinese fir plantation soils (SCP and TCP) exhibited a higher level of amino acid, carboxylic acids and phenolic acids than those from the first generation plantation soils (FCP). The AWCD values of amine showed no significant differences among different planting rotations soils. However, the sum of AWCD values of amino acids, carboxylic acids and phenolic compounds were higher significantly in the SCP and TCP soils than FCP soils (Fig. 4), indicated that acids gradually became predominant in the continuous monoculture plantation soils.

### The correlation analysis between microbial community diversity and soil properties

The correlation analysis between microbial community diversity and soil properties was showed in Table 4. The results showed that all the soil physical and chemical property indexes were positively correlated with the diversity indexes except soil pH. There was extremely significant correlation between diversity index and TOC and AN. In contrast, the C/N ratio showed a significant negative correlation with diversity index, suggesting that soil C and N contents play an important role on soil microbial diversity.

**Table 4:** Correlation analysis of microbial community diversity and soil properties.

	Simpson index	Shannon index	Richness index	McIntosh index
pH	−0.395	−0.347	−0.384	−0.29
TOC/(g·kg^−1^)	0.948^**^	0.977^**^	0.965^**^	0.983^**^
TN/(g·kg^−1^)	0.859^*^	0.8062^*^	0.725	0.870^*^
AN/(mg·kg^−1^)	0.953^**^	0.937^**^	0.918^**^	0.892^**^
TP/(g·kg^−1^)	0.734	0.887^*^	0.891^*^	0.702
AP/(mg·kg^−1^)	0.911^**^	0.766	0.721	0.634
TK/(mg·kg^−1^)	0.862^*^	0.810	0.715	0.744
AK/(mg·kg^−1^)	0.537	0.682	0.705	0.725
C/N	−0.934^**^	−0.927^**^	−0.919^**^	−0.971^**^

Note: ^*^means *P* < 0. 05, significant correlation; ^**^means *P* < 0. 01, extremely significant correlation.

## Discussion

Soil microorganisms are vital for forest ecological system, which have a huge impact in material circulation and energy flow. Different forest management will change the soil microbial community structure and metabolism, resulting in negative feedback on plant growth^24^. In this study, we demonstrated that soil microbial community structure and metabolic activity significantly degenerated in successive rotations of Chinese fir plantations.

In this study, three important conclusions were found. The first conclusion was that consecutive monoculture of Chinese fir plantations resulted in the descending of soil microbial community diversity and great change of soil microbial community structure. The total content of PLFA biomarkers was highest in FCP, followed by SCP, and TCP was the least detected. The result was consistent with previous researches. Liu *et al*. found that monoculture of Chinese fir resulted in the decrease of the soil microbial biomass, and broad-leaved tree mixed planting with Chinese fir could improve the balance of soil microflora^5^. Besides that, the soil microbial community structure also changed in successive rotations of Chinese fir plantations. The amount of bacteria PLFAs changed from 83.99 ± 1.47 (ug·g^−1^) in FCP to 69.72 ± 0.86 (ug·g^−1^) in TCP, whereas the amount of Fungi PLFAs changed from 10.92 ± 0.11 (ug·g^−1^) in FCP to 16.39 ± 0.23 (ug·g^−1^) in TCP soils. The F/B ratio significantly increased in SCP and TCP soils. The F/B ratio is an important indication of soil environment conditions. Generally, bacteria will increase when soil nutrients are enough. Conversely, fungi will increase when soil nutrient decline, because fungi are more able to adapt to the bad soil environment^25^. In this study, we found that in FCP soil, adequate soil nutrition resulted in the reproduction of bacteria. However, in SCP and TCP soil, nutrient content decrease resulted in the reproduction of fungi. Similar result was also reported by Zhao *et al*. in which soil fungal diversity increased in *Eucalyptus* monocultures^26^.

The second conclusion was that the catabolic activity of soil microbial community decreased in consecutive monoculture of Chinese fir plantations. The FCP soil showed the highest utilization rate of carbon source, whereas the TCP soil showed the lowest utilization rate (FCP > SCP > TCP). The sum of AWCD values of amino acids, carboxylic acids and phenolic compounds were higher significantly in the SCP and TCP soils than FCP soils, indicated that acids gradually became predominant in the continuous monoculture plantation soils. Many scholars have focused on the phenolic acids and their ecological effect on soil microflora. Our results are consistent with those of Wu *et al*., who revealed that the consumption of acid carbon substrates in the consecutively monocultured *Rehmannia glutinosasoil* was significantly greater than that in the newly planted soil^12^. Furthermore, Xia *et al*. suggest that cyclic dipeptide is a highly active allelochemical with a phytotoxic effect that limits soil microbial catabolic activity in the replanted Chinese fir tree ecosystem^27^. Trial evidence obtained from laboratory tests and field experiments support the fact that any increasing concentrations of phenolic acids with high bioactive dosage could be degraded by soil microorganisms, and this process might mediate the changes in microbial diversity in the rhizosphere. More attention should be paid to the other compounds involved, such as amino acids and fatty acids.

The third conclusion was that soil nutrient content play an important role in shaping microbial communities, and soil C/N ratio was one of the most important factors to soil microbial diversity. Soil C/N ratio is an important index of the physical and chemical properties, which affect plant growth and soil microbial balance. Our resulted showed that there exist significantly negatively correlation between soil microbial community diversity and C/N ratio. This result is also consistent with previous studies^28^. For example, Lucas-Borja *et al*. pointed out that the differences in the biomass and structure of the soil microbial community are related to changes in the soil C/N ratio and pH^29^. A higher ratio usually suggests a high proportion of fungi compared to bacteria^30^. The ratio of microbial C/N in FCP was significantly lower than that in SCP and TCP plantations. This indicated that the proportion of bacteria increased in long-term monoculture plantations. Furthermore, the effects of spatial change and seasonal variation on soil nutrient content are also very important^31–34^. Seasonal shifts affect soil microbial community composition by changing the soil physicochemical properties. The soil sampling in this study was only carried out in March 2016, based on our previous research results that, the soil physicochemical properties have no significant differences among different seasons in Chinese fir plantations^35^.

On the whole, our conclusions indicated that soil microbial community structure and metabolic activity significantly degenerated in successive rotations of Chinese fir plantations. The data generated in this study cannot discriminate among pathogenic and non-pathogenic fungus. However, it imply that imbalance of soil microbial community may cause the CMP of Chinese fir. Both the PLFA and CLPP have limitations in detection of soil microbial community. With the development of soil microbial ecology, high-throughput DNA sequencing technology or metagenomics technology have moved into the mainstream. Furthermore, the seasonal variation plays an important role in vegetation diversity and soil physical and chemical characters. In future works, we will use high-throughput sequencing and terminal restriction fragment length polymorphism method (T-RFLP) to identify the harmful and beneficial microorganisms in Chinese fir plantations. The hypothesis that soil microbial community structure would vary with seasonal shifts in Chinese fir plantations will also be examined.

## Methods

### Experiment site

The experiment site was chosen at Youxi National Forest Farm, Fujian Province, China, an 21.5 km^2^ forested area in the mid-subtropical region (latitude 25°48′N–26°24′N and longitude 117°48′E–118°36′E). It’s one of main producing areas of Chinese fir and has typical soil (yellow-red soil) and typical climate of Southern China. The annual average temperature is 18.9 °C. The extreme high temperature is 40.3 °C and the extreme low temperature is −7.8 °C. The annual mean relative humidity is 83.0% and the annual precipitation reaches 1599.6 mm.

## Soil sampling

Three different Chinese fir plantations were selected. The first Chinese fir rotation plantation (FCP) was established in 1988, and the second rotation plantation (SCP) was established in 1989, and the third rotation plantation (TCP) was established in 1992. We established three soil sampling plots (20 m × 20 m) from FCP, SCP and TCP in March 2016. Soil samples were randomly collected from 0–20 cm depths in each plot using a soil core sampler. The method of field sampling and soil physicochemical properties determination was according to Wu *et al*.^7^.

## Experimental methods

The phospholipid fatty acid (PLFAs) were extracted according to Zelles *et al*.^36^. We used 450GC/240MS system (Varian, Inc. USA) to determine the concentration of PLFAs following the procedure described by Patra *et al*.^37^. The PLFAs classification was according to Joergensen and Wichern^38–41^. BIOLOG Eco Microplate^TM^ system (BIOLOG Inc., CA, USA) was used according to Ma *et al*.^42^. Automatic microplate reader (SpectraMax M5, USA) was used to record the average well-color development (AWCD).

## Electronic supplementary material

Supplementary Information
