# Supplementary material for: Soil microbial community structure and catabolic activity are significantly degenerated in successive rotations of Chinese fir plantations
Source: Sci Rep. 2017 Jul 27;7:6691. doi: 10.1038/s41598-017-06768-x (PMC5532251; doi:10.1038/s41598-017-06768-x)
Supplement: Supplementary file 1 — Supplementary Information [file 41598_2017_6768_MOESM1_ESM.pdf]

Soil microbial community structure and catabolic activity are significantly degenerated in successive rotations of Chinese fir plantations

Zeyan Wu[1,2,3], Jianjuan Li[4,+], Jie Zheng[4], Jinfu Liu[4], Shuying Liu[1], Wenxiong Lin[1,2,3,*], Chengzhen Wu[4,*]

1. Life Sciences College of Fujian Agriculture and Forestry University, Fujian350002, China;

2. Fujian Provincial Key Laboratory of Agroecological Processing and Safety Monitoring, School of Life Sciences, Fujian Agriculture and Forestry University, Fuzhou 350002, China;

3. Key Laboratory of Crop Ecology and Molecular Physiology (Fujian Agriculture and Forestry University), Fujian Province University, Fuzhou 350002, China;

4. Forestry College of Fujian Agriculture and Forestry University, Fujian350002, China.

*corresponding author: wenxiong1810@163.com; chengzhenwu110@126.com

+ this authors contributed equally to this work

The optical density OD at 590nm change with incubation time (Day 1)

Application: Tecan i-control Tecan i-control , 1.11.1.0
Device: infinite 200Pro Serial number: 1412005097 Serial number of connected stacker:
Firmware: V_3.37_07/12_Infinite (JulMAI, V_3.37_07/12_Infinite (Jul 20 2012/13.56.47)
System NLDX-PC
User nldx-PC\nldx
Plate Greiner 96 Flat Bottom Transparent Polystyrene Cat. No.: 655101/655161/655192
Plate-ID (Stacker)
Label: Label1
Mode Absorbance
Wavelength 590 nm
Bandwidth 9 nm
Number of Flashes 25
Settle Time 0 ms
Start Tin2016/3/30 13:11:27

Supplementary Table S1. The optical density OD at 590nm change with incubation time (Day 1)

	1	2	3	4	5	6	7	8	9	10	11	12
A	0.0495	0.0715	0.0492	0.0512	0.0579	0.0551	0.1518	0.0684	0.0508	0.076	0.0501	0.0474
B	0.0994	0.1214	0.1142	0.0765	0.0904	0.1236	0.0773	0.0854	0.0648	0.1101	0.067	0.0537
C	0.3155	0.048	0.0472	0.0773	0.0793	0.0449	0.0456	0.0453	0.1184	0.0602	0.0456	0.0836
D	0.0668	0.0529	0.1999	0.0467	0.0726	0.0686	0.0461	0.0489	0.1162	0.047	0.0545	0.1087
E	0.0802	0.1445	0.0535	0.0653	0.0987	0.0776	0.6944	0.0575	0.0502	0.0859	0.0907	0.0512
F	0.0848	0.0779	0.0446	0.0773	0.0753	0.0734	0.0821	0.0564	0.0885	0.0994	0.1001	0.0688
G	0.0611	0.0511	0.0649	0.0861	0.0686	0.0597	0.0512	0.0455	0.0493	0.0539	0.0661	0.0575
H	0.0534	0.0979	0.0513	0.0498	0.0795	0.0574	0.0686	0.1118	0.0619	0.0713	0.0695	0.0514

The optical density OD at 590nm change with incubation time (Day 2)

Application: Tecan i-control	Tecan i-control , 1.11.1.0	
Device: infinite 200Pro	Serial number: 1412005097	Serial number of connected stacker:
Firmware: V_3.37_07/12_Infinite (Jul 20 20	MAI, V_3.37_07/12_Infinite (Jul 20 2012/13.56.47)	
System	NLDX-PC	
User	nldx-PC\nldx	
Plate	Greiner 96 Flat Bottom Transparent Polystyrene Cat. No.: 655101/655161/655192 [GRE96ft.pdfx]	
Plate-ID (Stacker)		
Label: Label1		
Mode	Absorbance	
Wavelength	590 nm	
Bandwidth	9 nm	
Number of Flashes	25	
Settle Time	0 ms	
Start Time 2016/3/31 12:33:52		

Supplementary Table S2. The optical density OD at 590nm change with incubation time (Day 2)

	1	2	3	4	5	6	7	8	9	10	11	12
A	0.0489	0.0657	0.0525	0.0518	0.0486	0.0652	0.5915	0.4109	0.0521	0.0594	0.0516	0.0572
B	0.7902	0.1208	1.0191	0.1216	0.0782	0.1113	0.7485	0.1028	0.7909	0.0979	0.0641	0.6421
C	1.0588	0.0563	0.049	0.356	0.0757	0.0462	0.1418	0.2912	0.6247	0.0616	0.0466	0.4175
D	0.4808	0.1308	0.1878	0.0646	0.1163	0.6629	0.0445	0.0492	0.1509	0.152	0.4602	0.0927
E	0.0867	0.5877	0.0544	0.0665	0.0908	0.0932	0.7282	0.0556	0.0727	0.1035	0.0904	0.0828
F	0.1002	0.7041	0.0469	0.1204	0.0706	0.0729	0.0945	0.0939	0.0929	0.9831	0.3878	0.0834
G	0.0695	0.0718	0.0642	0.3973	0.0695	0.0611	0.0497	0.0454	0.0688	0.0649	0.0791	0.3162
H	0.1054	0.1369	0.0544	0.0895	0.0756	0.0714	1.8805	0.1124	0.0615	0.0679	0.2221	0.3673

The optical density OD at 590nm change with incubation time (Day 3)

Application: Tecan i-control	Tecan i-control , 1.11.1.0	
Device: infinite 200Pro	Serial number: 1412005097	Serial number of connected stacker:
Firmware: V_3.37_07/12_Infinite (Jul 20 20	MAI, V_3.37_07/12_Infinite (Jul 20 2012/13.56.47)	
System	NLDX-PC	
User	nldx-PC\nldx	
Plate	Greiner 96 Flat Bottom Transparent Polystyrene Cat. No.: 655101/655161/655192	
Plate-ID (Stacker)		
Label: Label1		
Mode	Absorbance	
Wavelength	590 nm	
Bandwidth	9 nm	
Number of Flashes	25	
Settle Time	0 ms	
Start Time 2016/4/1 12:29:08		

Supplementary Table S3. The optical density OD at 590nm change with incubation time (Day 3)

	1	2	3	4	5	6	7	8	9	10	11	12
A	0.0498	0.0604	0.058	0.3244	0.0471	0.063	0.5691	0.5206	0.0497	0.053	0.0625	0.082
B	1.075	0.1153	1.1181	0.433	0.0782	0.1068	1.0845	0.1299	1.2627	0.093	0.6725	1.4814
C	1.1221	0.0555	0.0478	0.6391	0.2771	0.0446	0.4647	0.5514	0.925	0.0522	0.0463	0.6437
D	0.6989	0.8118	0.3201	0.0887	0.1083	0.8278	0.0499	0.0478	0.1241	0.7939	0.4773	0.0783
E	0.0783	0.7662	0.058	0.0791	0.3298	0.1498	0.7535	0.0552	0.3797	0.5629	0.0795	0.5947
F	0.1844	0.8813	0.047	0.2295	0.0805	0.09	0.0788	0.1025	0.0986	1.2471	0.5187	0.0943
G	0.078	0.072	0.073	0.5962	0.0776	0.0579	0.0492	0.0453	0.067	0.1101	0.0844	0.5581
H	0.3621	0.2427	0.0551	0.2628	0.0682	0.0817	2.0064	0.1077	0.0648	0.0632	0.7789	0.5619

The optical density OD at 590nm change with incubation time (Day 4)

Application: Tecan i-control	Tecan i-control , 1.11.1.0	
Device: infinite 200Pro	Serial number: 1412005097	Serial number of connected stacker:
Firmware: V_3.37_07/12_Infinite (Jul 20 20	MAI, V_3.37_07/12_Infinite (Jul 20 2012/13.56.47)	
System	NLDX-PC	
User	nldx-PC\nldx	
Plate	Greiner 96 Flat Bottom Transparent Polystyrene Cat. No.: 655101/655161/655192	
Plate-ID (Stacker)		
Label: Label1		
Mode	Absorbance	
Wavelength	590 nm	
Bandwidth	9 nm	
Number of Flashes	25	
Settle Time	0 ms	
Start Time 2016/4/2 12:36:28		

Supplementary Table S4. The optical density OD at 590nm change with incubation time (Day 4)

	1	2	3	4	5	6	7	8	9	10	11	12
A	0.05	0.0587	0.062	0.7757	0.0475	0.076	0.5694	0.8352	0.0505	0.0511	0.0671	0.2836
B	1.4326	0.1072	1.5836	0.617	0.0842	0.1006	1.4596	0.3858	1.5634	0.0892	1.4498	1.8908
C	1.1278	0.0629	0.0485	1.0148	0.7128	0.0458	0.6947	1.0012	1.1535	0.0575	0.0469	0.7051
D	0.9215	1.1537	0.5896	0.169	0.1377	1.1744	0.0605	0.0479	0.1553	1.23	0.7938	0.187
E	0.0743	0.8935	0.0616	0.1388	0.6032	0.3523	0.7382	0.0537	0.6768	0.8956	0.1275	1.2487
F	0.3331	1.0209	0.0482	0.6679	0.2648	0.2801	0.089	0.1606	0.1101	1.5658	0.5527	0.1318
G	0.0981	0.1036	0.1348	0.8423	0.4695	0.0609	0.0498	0.0463	0.0994	0.1145	0.1107	0.7091
H	0.5237	0.3511	0.0562	0.5536	0.0763	0.1224	2.0548	0.0485	0.068	0.0737	0.9026	0.6866

The optical density OD at 590nm change with incubation time (Day 5)

Application: Tecan i-control	Tecan i-control , 1.11.1.0	
Device: infinite 200Pro	Serial number: 1412005097	Serial number of connected stacker:
Firmware: V_3.37_07/12_Infinite (Jul 20 20	MAI, V_3.37_07/12_Infinite (Jul 20 2012/13.56.47)	
System	NLDX-PC	
User	nldx-PC\nldx	
Plate	Greiner 96 Flat Bottom Transparent Polystyrene Cat. No.: 655101/655161/655192	
Plate-ID (Stacker)		
Label: Label1		
Mode	Absorbance	
Wavelength	590 nm	
Bandwidth	9 nm	
Number of Flashes	25	
Settle Time	0 ms	
Start Time 2016/4/3 12:46:44		

Supplementary Table S5. The optical density OD at 590nm change with incubation time (Day 5)

	1	2	3	4	5	6	7	8	9	10	11	12
A	0.051	0.0597	0.0671	0.6175	0.0478	0.1439	0.5596	0.5227	0.0497	0.051	0.0749	0.2519
B	0.9789	0.104	1.124	0.799	0.0936	0.0997	1.4038	0.4717	1.5557	0.0891	1.5403	1.9098
C	1.2072	0.0563	0.0492	0.8585	0.7885	0.0465	0.731	0.4798	1.3287	0.0528	0.0478	0.7627
D	1.0208	1.1422	0.2948	0.2102	0.1168	1.0738	0.0625	0.0483	0.161	1.0591	0.4901	0.2024
E	0.0667	1.0026	0.0603	0.1346	0.6634	0.2649	0.7346	0.0539	0.6372	1.1063	0.1187	1.3099
F	0.435	1.2583	0.0493	0.6383	0.2137	0.6035	0.0672	0.1043	0.0783	1.5404	0.5822	0.1251
G	0.1183	0.0769	0.1854	0.6336	0.6876	0.0623	0.0497	0.0468	0.075	0.1094	0.1114	0.7205
H	0.5575	0.2273	0.0569	0.3357	0.0772	0.1324	1.8354	0.0493	0.0751	0.0784	0.7953	0.7265

The optical density OD at 590nm change with incubation time (Day 6)

Application: Tecan i-control	Tecan i-control , 1.11.1.0	
Device: infinite 200Pro	Serial number: 1412005097	Serial number of connected stacker:
Firmware: V_3.37_07/12_Infinite (Jul 20 20	MAI, V_3.37_07/12_Infinite (Jul 20 2012/13.56.47)	
System	NLDX-PC	
User	nldx-PC\nldx	
Plate	Greiner 96 Flat Bottom Transparent Polystyrene Cat. No.: 655101/655161/655192 [GRE96ft.pdfx]	
Plate-ID (Stacker)		
Label: Label1		
Mode	Absorbance	
Wavelength	590 nm	
Bandwidth	9 nm	
Number of Flashes	25	
Settle Time	0 ms	
Start Time 2016/4/4 12:25:07		

Supplementary Table S6. The optical density OD at 590nm change with incubation time (Day 6)

	1	2	3	4	5	6	7	8	9	10	11	12
A	0.0547	0.0626	0.1068	1.252	0.0494	0.3262	0.5492	1.3413	0.0498	0.0519	0.1353	0.3673
B	1.6527	0.1042	1.8825	0.9703	0.1184	0.1074	1.7623	1.3116	1.5576	0.0925	1.922	2.0557
C	1.2863	0.0705	0.0493	1.4558	1.0031	0.0467	1.0664	0.8946	1.4701	0.0518	0.0484	0.8031
D	1.1142	1.5074	0.8603	0.5197	0.2192	1.5034	0.0638	0.0491	0.4985	1.2065	0.8335	0.3414
E	0.0886	1.0734	0.0627	0.2601	0.9752	0.5789	0.7435	0.0646	0.7773	1.277	0.1161	1.5575
F	0.5713	1.3899	0.0501	0.8354	0.4141	1.1701	0.0842	0.1445	0.094	1.5959	0.6155	0.1825
G	0.2262	0.1285	0.316	1.0031	1.3099	0.0633	0.05	0.0472	0.0914	0.1108	0.1669	0.7445
H	0.837	0.3195	0.0576	0.4495	0.0859	0.1917	1.8246	0.0502	0.088	0.0698	0.9189	0.7599

The optical density OD at 590nm change with incubation time (Day 7)

Application: Tecan i-control Tecan i-control , 1.11.1.0

Device: infinite 200Pro Serial number: 1412005097 Serial number of connected stacker:

Firmware: V_3.37_07/12_Infinite (Jul 20 20 MAI, V_3.37_07/12_Infinite (Jul 20 2012/13.56.47)

System NLDX-PC

User nldx-PC\nldx

Plate Greiner 96 Flat Bottom Transparent Polystyrene Cat. No.: 655101/655161/655192

Plate-ID (Stacker)

Label: Label1

Mode Absorbance

Wavelength 590 nm

Bandwidth 9 nm

Number of Flashes 25

Settle Time 0 ms

Start Time 2016/4/5 12:26:52

Supplementary Table S7. The optical density OD at 590nm change with incubation time (Day 7)

	1	2	3	4	5	6	7	8	9	10	11	12
A	0.0522	0.064	0.1273	0.618	0.0485	0.3357	0.5625	0.8066	0.0489	0.0593	0.2206	0.3769
B	1.3142	0.0987	1.4732	1.1072	0.1359	0.0944	1.3602	0.8561	1.531	0.0867	1.5464	2.1579
C	1.3458	0.0658	0.0506	1.0836	1.0068	0.0475	1.2612	0.8328	1.6411	0.0552	0.0483	0.8567
D	1.1163	1.4427	0.5278	0.417	0.2369	1.3831	0.0598	0.0482	0.8065	1.0876	0.6636	0.3717
E	0.0734	1.0983	0.0643	0.2114	1.0202	0.5115	0.7447	0.0524	0.7008	1.3708	0.1185	1.335
F	0.5985	1.343	0.0514	0.7634	0.4257	1.5413	0.0699	0.1407	0.0837	1.5996	0.6374	0.1904
G	0.2309	0.1192	0.389	0.9433	1.1454	0.0638	0.05	0.0475	0.0954	0.115	0.1552	0.842
H	0.828	0.2375	0.058	0.4169	0.0992	0.2426	1.819	0.0496	0.098	0.0874	0.8745	0.8023

Sample Report
Sample ID: 1
Operator: zxy
Instrument ID: Varian GC/MS #1
Acquisition Date: 4/7/2016 15:33 PM
Method: D:\2015data\lina20151029\20140712.mth
Inj. Sample Notes: None

Supplementary Table S8. PLFA determination of microbial community composition （FCP）

Peaks: 294("Peak Number"	RT (min)	Area	CAS Number	Name
1	5.259	2.87E+06	151-10-0	Benzene, 1,3-dimethoxy-
2	5.365	25416	17721-95-8	Piperidine, 2,6-dimethyl-1-nitroso-
3	5.431	2.13E+06	None	Oxalic acid, isobutyl nonyl ester
4	5.514	162884	13131-19-6	N-Methyl-9-aza-tricyclo[6.2.2.0(2,7)]dod
5	5.58	67083	72439-85-1	3,5-Dibutoxy-1,1,1,7,7,7-hexamethyl-3,5-
6	5.701	60052	1746-13-0	Benzene, (2-propenyloxy)-
7	5.772	41337	877-44-1	Benzene, 1,2,4-triethyl-
8	5.814	103406	37385-06-1	Pentanamide, N-1H-purin-6-yl-
9	5.894	88141	None	Oxalic acid, isobutyl nonyl ester
10	5.951	69287	56298-75-0	1H-Indene, 1-ethyl-2,3-dihydro-1-methyl-
11	5.976	105947	None	Oxalic acid, allyl nonyl ester
12	6.05	131283	540-97-6	Cyclohexasiloxane, dodecamethyl-
13	6.093	46326	None	1-(4-Isopropylphenyl)-3-(tetrahydrofuryl
14	6.166	1.46E+06	None	N-HEXACOSANE
15	6.361	609297	90-12-0	Naphthalene, 1-methyl-
16	6.504	377474	90-12-0	Naphthalene, 1-methyl-
17	6.583	113108	29812-79-1	Hydroxylamine, O-decyl-
18	6.629	64935	56599-40-7	1,3-Dioxane, 4-(hexadecyloxy)-2-pentadec
19	6.685	108691	None	Limonen-6-ol, pivalate
20	6.747	231652	74630-39-0	1-Undecene, 4-methyl-

21	6.924	757678	5345-42-6	Benzene, 1-methoxy-3-methyl-2-nitro-
22	7.013	1.33E+06	None	N-HEXACOSANE
23	7.068	185712	131147-55-2	5-Ethenyl-5-(1-methyl-3-butenyl)-hexahyd
24	7.147	199790	30889-32-8	1,3-Dioxolane-4-methanol, 2-pentadecyl-,
25	7.233	222883	1127-76-0	Naphthalene, 1-ethyl-
26	7.362	627578	581-40-8	Naphthalene, 2,3-dimethyl-
27	7.438	282992	4630/7/3	Naphthalene, 1,2,3,5,6,7,8,8a-octahydro-
28	7.505	659624	575-41-7	Naphthalene, 1,3-dimethyl-
29	7.555	445700	575-41-7	Naphthalene, 1,3-dimethyl-
30	7.694	251921	98419-10-4	4,4-Dimethyl-3-(3-methylbut-2-enylidene)
31	7.744	269219	575-41-7	Naphthalene, 1,3-dimethyl-
32	7.829	498240	719-22-2	2,5-Cyclohexadiene-1,4-dione, 2,6-bis(1,
33	7.925	360546	None	1-ETHYLNAPHTHALENE
34	8.015	87585	55320-02-0	9,12,15-Octadecatrienoic acid, 2,3-bis(a
35	8.106	179602	None	N-HEXACOSANE
36	8.154	52957	540-97-6	Cyclohexasiloxane, dodecamethyl-
37	8.223	205860	644-08-6	1,1'-Biphenyl, 4-methyl-
38	8.268	1.24E+06	138345-00-3	7,9-Di-tertbutyl-1-oxaspiro[4,5]deca-6,9
39	8.355	131219	644-08-6	1,1'-Biphenyl, 4-methyl-
40	8.406	235312	111-82-0	Dodecanoic acid, methyl ester
41	8.503	137170	None	3-(2-Methyl-propenyl)-1H-indene
42	8.526	183163	192823-15-7	Decane, 2,3,5,8-tetramethyl-
43	8.704	388906	620-47-3	Benzene, 1-methyl-3-(phenylmethyl)-
44	8.761	256212	2245-38-7	Naphthalene, 1,6,7-trimethyl-
45	8.854	162917	829-26-5	Naphthalene, 2,3,6-trimethyl-
46	8.934	197012	13151-91-2	Tridecane, 6-cyclohexyl-
47	9.046	216612	None	3-(2-Methyl-propenyl)-1H-indene
48	9.131	142600	2131-42-2	Naphthalene, 1,4,6-trimethyl-
49	9.276	165672	2500-59-6	Oxiraneoctanoic acid, 3-octyl-, methyl e
50	9.358	184641	53800-02-5	Trifluoroacetic acid,n-tridecyl ester
51	9.463	1.16E+06	None	N-HEXACOSANE

52	9.606	167041	13151-91-2	Tridecane, 6-cyclohexyl-
53	9.669	98974	556-68-3	Cyclooctasiloxane, hexadecamethyl-
54	9.712	124959	1724-39-6	Cyclododecanol
55	9.825	76654	5129-65-7	Dodecanoic acid, 10-methyl-, methyl este
56	10.053	89256	None	Epicedrol
57	10.15	300148	112-95-8	Eicosane
58	10.418	117456	None	Oxalic acid, allyl octadecyl ester
59	10.544	200723	10544-96-4	Octadecane, 6-methyl-
60	10.596	60712	None	Phthalic acid, methyl octyl ester
61	10.687	79063	36653-82-4	1-Hexadecanol
62	10.778	145240	732-26-3	Phenol, 2,4,6-tris(1,1-dimethylethyl)-
63	10.82	232565	5129-58-8	Tridecanoic acid, 12-methyl-, methyl est
64	10.917	71500	None	Methoxyacetic acid, 3-tridecyl ester
65	11.023	1.24E+06	None	N-HEXACOSANE
66	11.331	82632	1724-39-6	Cyclododecanol
67	11.36	43251	None	2-Amino-6,7-dimethyl-5,6,7,8-tetrahydro-
68	11.429	765384	5129-58-8	Tridecanoic acid, 12-methyl-, methyl est
69	11.723	214786	630-06-8	Hexatriacontane
70	11.857	87849	54105-67-8	Heptadecane, 2,6-dimethyl-
71	11.967	134028	2490-48-4	1-Hexadecanol, 2-methyl-
72	12.084	250754	930-02-9	Octadecane, 1-(ethenyloxy)-
73	12.212	469589	None	Methoxyacetic acid, 3-tridecyl ester
74	12.321	83768	2825-81-2	2-Hexadecenoic acid, methyl ester, (E)-
75	12.514	2.16E+06	7132-64-1	Pentadecanoic acid, methyl ester
76	12.656	887559	5129-66-8	Tetradecanoic acid, 12-methyl-, methyl e
77	12.724	1.34E+06	None	N-HEXACOSANE
78	12.824	744196	None	N-HEXACOSANE
79	13.071	163704	1724-39-6	Cyclododecanol
80	13.163	453606	7132-64-1	Pentadecanoic acid, methyl ester
81	13.232	54365	112-39-0	Hexadecanoic acid, methyl ester
82	13.283	64191	55682-91-2	Heptacosanoic acid, methyl ester

83	13.379	90238	54153-31-0	2-(2-Diethylamino-ethoxy)-fluoren-9-one
84	13.459	245048	502-69-2	2-Pentadecanone, 6,10,14-trimethyl-
85	13.52	106562	14852-31-4	2-Hexadecanol
86	13.721	65315	None	Oxalic acid, dodecyl neopentyl ester
87	13.865	440229	84-69-5	1,2-Benzenedicarboxylic acid, bis(2-meth
88	13.918	194193	2416-20-8	Hexadecenoic acid, Z-11-
89	13.98	242757	15965-99-8	Oxirane, [(hexadecyloxy)methyl]-
90	14.186	259809	36653-82-4	1-Hexadecanol
91	14.291	2.06E+06	5129-60-2	Pentadecanoic acid, 14-methyl-, methyl e
92	14.372	25168	None	Limonen-6-ol, pivalate
93	14.497	1.21E+06	29812-79-1	Hydroxylamine, O-decyl-
94	14.586	991542	1120-25-8	9-Hexadecenoic acid, methyl ester, (Z)-
95	14.637	276679	29812-79-1	Hydroxylamine, O-decyl-
96	14.758	1.47E+06	1120-25-8	9-Hexadecenoic acid, methyl ester, (Z)-
97	14.854	33008	949-41-7	1H-Cyclopropa[l]phenanthrene,1a,9b-dihyd
98	14.969	6.34E+06	5129-60-2	Pentadecanoic acid, 14-methyl-, methyl e
99	15.102	32160	6386-38-5	Benzenepropanoic acid, 3,5-bis(1,1-dimet
100	15.192	98274	54410-98-9	1-Nonene, 4,6,8-trimethyl-
101	15.26	93550	949-41-7	1H-Cyclopropa[l]phenanthrene,1a,9b-dihyd
102	15.291	62120	None	Oxalic acid, decyl propyl ester
103	15.398	255762	None	N-HEXACOSANE
104	15.497	132634	None	Oxalic acid, allyl dodecyl ester
105	15.578	337103	117-84-0	Di-n-octylphthalate
106	15.654	674823	56875-67-3	7-Hexadecenoic acid, methyl ester, (Z)-
107	15.721	1.67E+06	2490-49-5	Hexadecanoic acid, 14-methyl-, methyl es
108	15.787	357794	10152-61-1	Cyclopropaneoctanoic acid, 2-hexyl-, met
109	15.85	780363	2490-49-5	Hexadecanoic acid, 14-methyl-, methyl es
110	15.916	279134	2825-81-2	2-Hexadecenoic acid, methyl ester, (E)-
111	16.09	1.43E+06	2490-49-5	Hexadecanoic acid, 14-methyl-, methyl es
112	16.176	151670	112-86-7	Erucic acid
113	16.243	808861	2490-49-5	Hexadecanoic acid, 14-methyl-, methyl es

114	16.284	1.32E+06	None	N-HEXACOSANE
115	16.487	583916	10152-61-1	Cyclopropaneoctanoic acid, 2-hexyl-, met
116	16.586	278582	128-37-0	Butylated Hydroxytoluene
117	16.69	234638	2529-64-8	Estra-1,3,5(10)-trien-17.beta.-ol
118	16.796	2.03E+06	2490-25-7	Heptadecanoic acid, 10-methyl-, methyl e
119	16.905	155944	56051-53-7	Cyclopropanebutanoic acid, 2-[[2-[[2-[(2
120	17.036	528256	55520-89-3	Hexadecanoic acid, trimethylsilyl ester
121	17.148	125568	18835-33-1	1-Hexacosene
122	17.251	443911	18772-36-6	Cyclodecasiloxane, eicosamethyl-
123	17.4	233348	14852-31-4	2-Hexadecanol
124	17.464	619757	2490-25-7	Heptadecanoic acid, 10-methyl-, methyl e
125	17.559	423380	112-80-1	Oleic Acid
126	17.668	265664	2566-89-4	5,8,11,14-Eicosatetraenoic acid, methyl
127	17.761	350500	None	Oleanitrile
128	17.821	673758	629-96-9	1-Eicosanol
129	17.877	246490	5129-61-3	Heptadecanoic acid, 16-methyl-, methyl e
130	17.963	3.97E+06	2462-85-3	9,12-Octadecadienoic acid, methyl ester
131	18.092	9.53E+06	1937-62-8	9-Octadecenoic acid, methyl ester, (E)-
132	18.18	2.23E+06	112-62-9	9-Octadecenoic acid (Z)-, methyl ester
133	18.26	718806	112-62-9	9-Octadecenoic acid (Z)-, methyl ester
134	18.354	1.02E+06	20290-84-0	9-Octadecenoic acid (Z)-, hexyl ester
135	18.536	3.90E+06	112-61-8	Octadecanoic acid, methyl ester
136	18.604	562871	22393-97-1	9-Hexadecenoic acid, 9-hexadecenyl ester
137	18.706	406607	None	2-Methyl-Z,Z-3,13-octadecadienol
138	18.781	390518	28813-61-8	2-Nonadecanone 2,4-dinitrophenylhydrazin
139	18.916	463511	55162-61-3	Tetracontane, 3,5,24-trimethyl-
140	19.02	289868	18835-33-1	1-Hexacosene
141	19.084	361254	19780-79-1	2-Hexyl-1-octanol
142	19.204	1.85E+06	55124-97-5	Octadecanoic acid, 17-methyl-, methyl es
143	19.246	823650	55124-97-5	Octadecanoic acid, 17-methyl-, methyl es
144	19.384	433417	55124-97-5	Octadecanoic acid, 17-methyl-, methyl es

145	19.486	895916	301-02-0	9-Octadecenamide, (Z)-
146	19.622	655207	18919-94-3	Tetracosamethyl-cyclododecasiloxane
147	19.691	280929	22393-97-1	9-Hexadecenoic acid, 9-hexadecenyl ester
148	19.786	1.40E+06	None	N-HEXACOSANE
149	19.87	260577	56700-76-6	9,12,15-Octadecatrienoic acid, 2-phenyl-
150	20.032	4.77E+06	10152-62-2	Cyclopropaneoctanoic acid, 2-octyl-, met
151	20.141	366924	None	.alpha.-d-Xylopyranoside, methyl-2,3,4-t
152	20.273	1.13E+06	1120-28-1	Eicosanoic acid, methyl ester
153	20.383	555458	641-85-0	Allopregnane
154	20.461	387349	18748-91-9	Octadecanoic acid, trimethylsilyl ester
155	20.542	219845	None	Cholestane, 2-formyl-3-(2-methylbenzylid
156	20.704	2.25E+06	505-54-4	Hexadecanedioic acid
157	20.76	618476	2566-89-4	5,8,11,14-Eicosatetraenoic acid, methyl
158	20.88	617341	2566-89-4	5,8,11,14-Eicosatetraenoic acid, methyl
159	21.004	368398	None	Tricyclo[20.8.0.0(7,16)]triacontane, 1(2
160	21.089	424587	None	Tricyclo[20.8.0.0(7,16)]triacontane, 1(2
161	21.172	257364	17673-25-5	Phorbol
162	21.227	348186	56554-77-9	13-Heptadecyn-1-ol
163	21.31	1.02E+06	6971-40-0	17-Pentatriacontene
164	21.375	76471	14852-31-4	2-Hexadecanol
165	21.465	1.67E+06	None	N-HEXACOSANE
166	21.603	514551	14852-31-4	2-Hexadecanol
167	21.681	501600	55334-01-5	Phenanthrene, 9-dodecyltetradecahydro-
168	21.761	161687	2490-48-4	1-Hexadecanol, 2-methyl-
169	21.864	526390	18919-94-3	Tetracosamethyl-cyclododecasiloxane
170	21.937	2.24E+06	1120-28-1	Eicosanoic acid, methyl ester
171	22.031	382790	None	N-HEXACOSANE
172	22.086	81795	29812-79-1	Hydroxylamine, O-decyl-
173	22.111	139441	74630-39-0	1-Undecene, 4-methyl-
174	22.187	365344	56847-05-3	9-Octadecen-12-ynoic acid, methyl ester
175	22.25	394860	1235-74-1	1-Phenanthrenecarboxylic acid, 1,2,3,4,4

176	22.327	96573	17453-58-6	3-Phorbinepropanoic acid, 9-acetyl-14-et
177	22.405	369273	14852-31-4	2-Hexadecanol
178	22.425	158109	18835-33-1	1-Hexacosene
179	22.531	3.20E+06	301-02-0	9-Octadecenamide, (Z)-
180	22.67	195657	301-02-0	9-Octadecenamide, (Z)-
181	22.69	511630	301-02-0	9-Octadecenamide, (Z)-
182	22.763	779279	301-02-0	9-Octadecenamide, (Z)-
183	22.935	1.31E+06	None	2-Methyl-Z,Z-3,13-octadecadienol
184	23.182	109315	None	Tricyclo[20.8.0.0(7,16)]triacontane, 1(2
185	23.202	98628	55521-22-7	9,12,15-Octadecatrienoic acid, 2,3-bis[(
186	23.3	823179	119-47-1	Phenol, 2,2'-methylenebis[6-(1,1-dimethy
187	23.407	541093	None	Tricyclo[20.8.0.0(7,16)]triacontane, 1(2
188	23.522	2.03E+06	None	Tricyclo[20.8.0.0(7,16)]triacontane, 1(2
189	23.607	537905	None	2-Methyl-Z,Z-3,13-octadecadienol
190	23.686	176130	14852-31-4	2-Hexadecanol
191	23.769	218762	None	12-Methyl-E,E-2,13-octadecadien-1-ol
192	23.787	195553	83040-97-5	3-(6,6-Dimethyl-5-oxohept-2-enyl)-cycloh
193	23.865	318051	61834-65-9	Allopregnane-3,7,11,20-tetra-one
194	23.993	1.18E+06	1472-93-1	Octadecanedioic acid, dimethyl ester
195	24.073	318293	1472-93-1	Octadecanedioic acid, dimethyl ester
196	24.154	93542	2629-11-0	d-Homo-24-nor-17-oxachola-20,22-diene-3,
197	24.238	427887	None	7,8-Epoxylanostan-11-ol, 3-acetoxy-
198	24.332	294351	None	Tricyclo[20.8.0.0(7,16)]triacontane, 1(2
199	24.363	60561	11094-59-0	Docosahexaenoic acid, 1,2,3-propanetriyl
200	24.432	299065	None	Phthalic acid, cyclohexyl 2-pentyl ester
201	24.452	103580	117-81-7	BIS(2-Ethylhexyl)phthalate
202	24.583	1.69E+06	629-96-9	1-Eicosanol
203	24.66	1.11E+06	19780-79-1	2-Hexyl-1-octanol
204	24.736	561906	56630-69-4	13-Docosenoic acid, methyl ester
205	24.831	233406	18835-33-1	1-Hexacosene
206	24.896	152530	18835-33-1	1-Hexacosene

207	24.942	157854	None	Pregnan-3,11-diol-20-one
208	25	118577	14852-31-4	2-Hexadecanol
209	25.016	81183	None	3-Methyl-4-(phenylthio)-2-prop-2-enyl-2,
210	25.036	64080	25328-53-4	Cholestan-3-one, cyclic 1,2-ethanediyl a
211	25.056	56976	None	3,19;14,15-Diepoxypregnan-20-one, 3,11,1
212	25.204	9.31E+06	117-81-7	BIS(2-Ethylhexyl)phthalate
213	25.233	3.58E+06	117-81-7	Di-2-ethylhexyl phthalate
214	25.262	5.28E+06	117-81-7	Di-2-ethylhexyl phthalate
215	25.467	203957	None	Phthalic acid, butyl undecyl ester
216	25.497	91481	119-07-3	1,2-Benzenedicarboxylic acid, decyl octy
217	25.518	227560	127611-84-1	3',8,8'-Trimethoxy-3-piperidyl-2,2'-bina
218	25.61	379503	None	Serverogenin acetate
219	25.705	373667	None	Tricyclo[20.8.0.0(7,16)]triacontane, 1(2
220	25.804	425226	7098-21-7	Tritetracontane
221	25.978	643602	18919-94-3	Tetracosamethyl-cyclododecasiloxane
222	26.074	389892	17453-58-6	3-Phorbinepropanoic acid, 9-acetyl-14-et
223	26.172	1.15E+06	19780-79-1	2-Hexyl-1-octanol
224	26.273	242364	30656-76-9	Cholesterol 3-O-[[2-acetoxy]ethyl]-
225	26.383	159533	77646-82-3	5H-Cyclopropa[3,4]benz[1,2-e]azulen-5-on
226	26.55	448344	None	9-Methyl-Z-10-tetradecen-1-ol acetate
227	26.641	507650	2433-97-8	Tricosanoic acid, methyl ester
228	26.707	426961	5353-25-3	Ethanol, 2-(9-octadecenyloxy)-, (Z)-
229	26.803	108429	1602-42-2	N-2,4-Dnp-L-arginine
230	26.826	58425	17453-58-6	3-Phorbinepropanoic acid, 9-acetyl-14-et
231	26.901	403717	56797-40-1	7-Hexadecenal, (Z)-
232	26.932	140598	None	Serverogenin acetate
233	26.963	145366	None	9,19-Cyclolanostan-3-ol, 24,24-epoxymeth
234	27.069	1.47E+06	None	9,19-Cyclolanostan-3-ol, 24,24-epoxymeth
235	27.234	1.31E+06	None	4,4,6a,6b,8a,11,11,14b-Octamethyl-1,4,4a
236	27.262	602676	None	4,4,6a,6b,8a,11,11,14b-Octamethyl-1,4,4a
237	27.296	1.42E+06	None	4,4,6a,6b,8a,11,11,14b-Octamethyl-1,4,4a

238	27.463	338780	None	4,4,6a,6b,8a,11,11,14b-Octamethyl-1,4,4a
239	27.533	59094	None	4,4,6a,6b,8a,11,11,14b-Octamethyl-1,4,4a
240	27.638	1.38E+06	None	Serverogenin acetate
241	27.739	3.03E+06	545-47-1	Lupeol
242	27.946	104101	None	3,9.beta.;14,15-Diepoxypregn-16-en-20-on
243	28.096	2.75E+06	2442-49-1	Tetracosanoic acid, methyl ester
244	28.281	94448	30365-65-2	Ergost-8(14)-en-3-ol, (3.beta.)-
245	28.333	269560	14852-31-4	2-Hexadecanol
246	28.411	105764	18641-57-1	Docosanoic acid, 1,2,3-propanetriyl este
247	28.441	51783	None	Ethyl iso-allocholate
248	28.496	161688	14852-31-4	2-Hexadecanol
249	28.526	95760	18835-33-1	1-Hexacosene
250	28.679	397442	17453-58-6	3-Phorbinepropanoic acid, 9-acetyl-14-et
251	28.722	131566	18835-33-1	1-Hexacosene
252	28.812	249729	14852-31-4	2-Hexadecanol
253	28.903	131230	25328-53-4	Cholestan-3-one, cyclic 1,2-ethanediyl a
254	28.926	57828	25328-53-4	Cholestan-3-one, cyclic 1,2-ethanediyl a
255	29.039	1.08E+06	122-62-3	Decanedioic acid, bis(2-ethylhexyl) este
256	29.234	1.03E+06	111-02-4	2,6,10,14,18,22-Tetracosahexaene, 2,6,10
257	29.489	769624	55373-89-2	Pentacosanoic acid, methyl ester
258	29.571	68309	25328-53-4	Cholestan-3-one, cyclic 1,2-ethanediyl a
259	29.591	143079	25328-53-4	Cholestan-3-one, cyclic 1,2-ethanediyl a
260	29.631	48114	55521-22-7	9,12,15-Octadecatrienoic acid, 2,3-bis[(
261	29.651	89877	None	3,9.beta.;14,15-Diepoxypregn-16-en-20-on
262	29.711	102445	None	3,9.beta.;14,15-Diepoxypregn-16-en-20-on
263	29.741	177642	None	3,9.beta.;14,15-Diepoxypregn-16-en-20-on
264	29.824	63816	105-92-0	Rhodopin
265	29.847	53446	537-40-6	Trilinolein
266	29.93	687860	22399-98-0	Docosanedioic acid, dimethyl ester
267	30.001	285264	22399-98-0	Docosanedioic acid, dimethyl ester
268	30.075	182297	22399-98-0	Docosanedioic acid, dimethyl ester

269	30.131	140624	None	3,9.beta.;14,15-Diepoxypregn-16-en-20-on
270	30.201	191243	77573-09-2	2-Butenoic acid, 2-methyl-, 1,1a,1b,4,4a
271	30.427	486931	None	Oxalic acid, allyl pentadecyl ester
272	30.55	185729	14852-31-4	2-Hexadecanol
273	30.634	39228	17453-58-6	3-Phorbinepropanoic acid, 9-acetyl-14-et
274	30.698	64118	17453-58-6	3-Phorbinepropanoic acid, 9-acetyl-14-et
275	30.725	20893	17453-58-6	3-Phorbinepropanoic acid, 9-acetyl-14-et
276	30.933	516465	5802-82-4	Hexacosanoic acid, methyl ester
277	30.987	54395	5802-82-4	Hexacosanoic acid, methyl ester
278	31.007	133869	5802-82-4	Hexacosanoic acid, methyl ester
279	31.073	87542	5802-82-4	Hexacosanoic acid, methyl ester
280	31.108	49965	5802-82-4	Hexacosanoic acid, methyl ester
281	31.138	31000	4657-58-3	Cycloartanol
282	31.179	99247	17453-58-6	3-Phorbinepropanoic acid, 9-acetyl-14-et
283	31.436	125597	6059-43-4	Dehydroergosterol 3,5-dinitrobenzoate
284	32.005	145481	42217-02-7	1-Chloroeicosane
285	33.249	420097	559-74-0	Friedelan-3-one
286	33.268	561915	559-74-0	Friedelan-3-one
287	33.883	83023	630-06-8	Hexatriacontane
288	34.62	44366	55682-92-3	Octacosanoic acid, methyl ester
289	34.935	27736	None	1-Cyclohexene, 1,3,3-trimethyl-2-(1-meth
290	34.954	21404	None	(7a-Isopropenyl-4,5-dimethyloctahydroind
291	36.115	14936	None	5-Cholestene-3-ol, 24-methyl-
292	36.14	35030	30365-65-2	Ergost-8(14)-en-3-ol, (3.beta.)-
293	36.509	77040	83-48-7	Stigmasterol
294	36.564	26290	83-48-7	Stigmasterol
Area Total:	1.78E+08			

PLFA determination of microbial community composition （SCP）

Sample Report

Sample ID: 2

Operator: zxy

Instrument ID: Varian GC/MS #1

Acquisition Date: 4/7/2016 18:38 PM

Method: D:\2015data\lina20151029\20140712.mth

Inj. Sample Notes: None

Supplementary Table S9. PLFA determination of microbial community composition （SCP）

Peaks: 227("Peak Number"	RT (min)	Area	CAS Number	Name
1	5.26	2.39E+06	151-10-0	Benzene, 1,3-dimethoxy-
2	5.365	27808	2432-99-7	Undecanoic acid, 11-amino-
3	5.434	2.10E+06	None	Oxalic acid, isobutyl nonyl ester
4	5.516	190366	13131-19-6	N-Methyl-9-aza-tricyclo[6.2.2.0(2,7)]dod
5	5.58	76001	72439-85-1	3,5-Dibutoxy-1,1,1,7,7,7-hexamethyl-3,5-
6	5.701	56212	1470-94-6	1H-Inden-5-ol, 2,3-dihydro-
7	5.773	33895	877-44-1	Benzene, 1,2,4-triethyl-
8	5.812	66560	None	Methoxyacetic acid, dodecyl ester
9	5.951	41475	56298-75-0	1H-Indene, 1-ethyl-2,3-dihydro-1-methyl-
10	5.978	50965	29812-79-1	Hydroxylamine, O-decyl-
11	6.051	25932	540-97-6	Cyclohexasiloxane, dodecamethyl-
12	6.168	1.35E+06	None	N-HEXACOSANE
13	6.361	586897	90-12-0	Naphthalene, 1-methyl-
14	6.505	305630	91-57-6	2-Methylnaphthalene
15	6.585	73285	17071-54-4	Hexyl octyl ether
16	6.629	36523	56599-40-7	1,3-Dioxane, 4-(hexadecyloxy)-2-pentadec
17	6.687	71469	544-76-3	Hexadecane
18	6.75	130697	195194-80-0	2-Piperidinone, N-[4-bromo-n-butyl]-

19	6.926	601856	5345-42-6	Benzene, 1-methoxy-3-methyl-2-nitro-
20	7.015	1.29E+06	None	Oxalic acid, isobutyl nonyl ester
21	7.071	146022	13151-80-9	Undecane, 5-cyclohexyl-
22	7.149	166505	2765/11/9	Pentadecanal-
23	7.235	165181	None	1-ETHYLNAPHTHALENE
24	7.361	548818	581-40-8	Naphthalene, 2,3-dimethyl-
25	7.505	679898	575-41-7	Naphthalene, 1,3-dimethyl-
26	7.558	375802	575-41-7	Naphthalene, 1,3-dimethyl-
27	7.695	211756	22198-47-6	4'-Ethoxy-2'-hydroxyoctanophenone
28	7.746	246417	None	1-ETHYLNAPHTHALENE
29	7.831	452741	719-22-2	2,5-Cyclohexadiene-1,4-dione, 2,6-bis(1,
30	7.927	210608	939-27-5	Naphthalene, 2-ethyl-
31	7.954	91766	74630-39-0	1-Undecene, 4-methyl-
32	8.019	55782	55401-63-3	9-Octadecenoic acid (Z)-, 2-(acetyloxy)-
33	8.106	124801	630-06-8	Hexatriacontane
34	8.271	1.96E+06	138345-00-3	7,9-Di-tertbutyl-1-oxaspiro[4,5]deca-6,9
35	8.359	107359	644-08-6	1,1'-Biphenyl, 4-methyl-
36	8.409	477270	111-82-0	Dodecanoic acid, methyl ester
37	8.507	226324	2027-17-0	Naphthalene, 2-(1-methylethyl)-
38	8.709	174497	620-47-3	Benzene, 1-methyl-3-(phenylmethyl)-
39	8.764	159453	829-26-5	Naphthalene, 2,3,6-trimethyl-
40	8.856	118152	829-26-5	Naphthalene, 2,3,6-trimethyl-
41	8.932	149718	13151-90-1	Tridecane, 5-cyclohexyl-
42	9.047	181565	14905-56-7	Tetradecane, 2,6,10-trimethyl-
43	9.13	163874	829-26-5	Naphthalene, 2,3,6-trimethyl-
44	9.279	207915	1115-01-1	Octadecanoic acid, 9,10-dihydroxy-, meth
45	9.359	170475	53800-02-5	Trifluoroacetic acid,n-tridecyl ester
46	9.464	947282	None	N-HEXACOSANE
47	9.608	153980	13151-91-2	Tridecane, 6-cyclohexyl-
48	9.714	127215	1724-39-6	Cyclododecanol

49	10.055	87717	None	Epicedrol
50	10.418	33334	None	Oxalic acid, allyl octadecyl ester
51	10.779	119720	732-26-3	Phenol, 2,4,6-tris(1,1-dimethylethyl)-
52	10.821	200878	5129-58-8	Tridecanoic acid, 12-methyl-, methyl est
53	11.019	275105	29812-79-1	Hydroxylamine, O-decyl-
54	11.062	118757	29812-79-1	Hydroxylamine, O-decyl-
55	11.238	46472	None	Methyl Z-11-tetradecenoate
56	11.434	1.01E+06	5129-58-8	Tridecanoic acid, 12-methyl-, methyl est
57	11.729	109262	29812-79-1	Hydroxylamine, O-decyl-
58	11.86	66218	2490-48-4	1-Hexadecanol, 2-methyl-
59	12.099	149459	14852-31-4	2-Hexadecanol
60	12.217	351719	14852-31-4	2-Hexadecanol
61	12.327	98413	None	Methyl Z-11-tetradecenoate
62	12.519	2.55E+06	7132-64-1	Pentadecanoic acid, methyl ester
63	12.659	1.06E+06	5129-66-8	Tetradecanoic acid, 12-methyl-, methyl e
64	12.724	668149	None	N-HEXACOSANE
65	12.823	155782	19780-79-1	2-Hexyl-1-octanol
66	12.891	199376	56875-67-3	7-Hexadecenoic acid, methyl ester, (Z)-
67	13.076	100278	2765/11/9	Pentadecanal-
68	13.166	473932	7132-64-1	Pentadecanoic acid, methyl ester
69	13.235	124205	112-39-0	Hexadecanoic acid, methyl ester
70	13.362	333551	102608-53-7	3,7,11,15-Tetramethyl-2-hexadecen-1-ol
71	13.471	277362	502-69-2	2-Pentadecanone, 6,10,14-trimethyl-
72	13.871	180220	84-78-6	1,2-Benzenedicarboxylic acid, butyl octy
73	13.936	391110	1120-25-8	9-Hexadecenoic acid, methyl ester, (Z)-
74	14.124	89573	102608-53-7	3,7,11,15-Tetramethyl-2-hexadecen-1-ol
75	14.193	64837	None	3-Trifluoroacetoxypentadecane
76	14.295	2.15E+06	5129-60-2	Pentadecanoic acid, 14-methyl-, methyl e
77	14.377	119116	10152-71-3	Cyclopropaneoctanoic acid, 2-[[2-[(2-eth
78	14.501	635684	56875-67-3	7-Hexadecenoic acid, methyl ester, (Z)-

79	14.591	1.88E+06	1120-25-8	9-Hexadecenoic acid, methyl ester, (Z)-
80	14.763	2.14E+06	1120-25-8	9-Hexadecenoic acid, methyl ester, (Z)-
81	14.867	133069	2416-20-8	Hexadecenoic acid, Z-11-
82	14.989	8.47E+06	5129-60-2	Pentadecanoic acid, 14-methyl-, methyl e
83	15.113	114858	6386-38-5	Benzenepropanoic acid, 3,5-bis(1,1-dimet
84	15.195	76019	14852-31-4	2-Hexadecanol
85	15.262	67353	112-80-1	Oleic Acid
86	15.393	188462	29812-79-1	Hydroxylamine, O-decyl-
87	15.507	47640	29812-79-1	Hydroxylamine, O-decyl-
88	15.583	167417	84-64-0	1,2-Benzenedicarboxylic acid, butyl cycl
89	15.657	811869	55044-54-7	11-Hexadecenoic acid, 15-methyl-, methyl
90	15.724	1.71E+06	2490-49-5	Hexadecanoic acid, 14-methyl-, methyl es
91	15.79	181495	42199-20-2	Cyclopropanepentanoic acid, 2-undecyl-,
92	15.853	518396	5129-66-8	Tetradecanoic acid, 12-methyl-, methyl e
93	15.923	220930	2825-81-2	2-Hexadecenoic acid, methyl ester, (E)-
94	16.093	1.46E+06	1731-92-6	Heptadecanoic acid, methyl ester
95	16.175	88080	2490-49-5	Hexadecanoic acid, 14-methyl-, methyl es
96	16.249	1.43E+06	2490-49-5	Hexadecanoic acid, 14-methyl-, methyl es
97	16.341	278432	56875-67-3	7-Hexadecenoic acid, methyl ester, (Z)-
98	16.49	616245	10152-61-1	Cyclopropaneoctanoic acid, 2-hexyl-, met
99	16.589	93049	128-37-0	Butylated Hydroxytoluene
100	16.622	54904	112-80-1	Oleic Acid
101	16.798	1.90E+06	2490-25-7	Heptadecanoic acid, 10-methyl-, methyl e
102	16.894	75470	54934-57-5	Heptadecanoic acid, 9-methyl-, methyl es
103	17.04	308080	55520-89-3	Hexadecanoic acid, trimethylsilyl ester
104	17.25	176707	18772-36-6	Cyclodecasiloxane, eicosamethyl-
105	17.47	571016	2490-25-7	Heptadecanoic acid, 10-methyl-, methyl e
106	17.564	247104	112-80-1	Oleic Acid
107	17.674	157569	59149-01-8	Methyl (Z)-5,11,14,17-eicosatetraenoate
108	17.769	314867	None	Oleanitrile

109	17.83	793538	629-96-9	1-Eicosanol
110	17.889	197585	5129-61-3	Heptadecanoic acid, 16-methyl-, methyl e
111	17.972	6.04E+06	2462-85-3	9,12-Octadecadienoic acid, methyl ester
112	18.1	1.06E+07	2716-53-2	2,3-Dihydroxypropyl elaidate
113	18.189	3.50E+06	112-62-9	9-Octadecenoic acid (Z)-, methyl ester
114	18.25	1.92E+06	150-86-7	Phytol
115	18.36	997358	112-62-9	9-Octadecenoic acid (Z)-, methyl ester
116	18.541	4.21E+06	112-61-8	Octadecanoic acid, methyl ester
117	18.604	583741	10152-62-2	Cyclopropaneoctanoic acid, 2-octyl-, met
118	18.703	180798	None	Z,E-2,13-Octadecadien-1-ol
119	18.794	307489	20576-58-3	2,6-Dodecadien-1-ol, 3,7,11-trimethyl-,
120	18.911	250822	930-02-9	Octadecane, 1-(ethenyloxy)-
121	19.015	166469	17367-08-7	Ethanol, 2-(9,12-octadecadienyloxy)-, (Z
122	19.091	160326	None	N-HEXACOSANE
123	19.2	1.97E+06	2490-19-9	Octadecanoic acid, 10-methyl-, methyl es
124	19.486	387613	14435-34-8	2-Octadecenoic acid, methyl ester
125	19.626	332213	18919-94-3	Tetracosamethyl-cyclododecasiloxane
126	19.688	71943	25328-53-4	Cholestan-3-one, cyclic 1,2-ethanediyl a
127	19.788	723505	630-06-8	Hexatriacontane
128	20.033	3.73E+06	10152-62-2	Cyclopropaneoctanoic acid, 2-octyl-, met
129	20.143	81505	2091-29-4	9-Hexadecenoic acid
130	20.277	493813	1120-28-1	Eicosanoic acid, methyl ester
131	20.467	216305	18748-91-9	Octadecanoic acid, trimethylsilyl ester
132	20.633	37422	19780-79-1	2-Hexyl-1-octanol
133	20.712	1.21E+06	505-54-4	Hexadecanedioic acid
134	20.772	1.60E+06	2566-89-4	5,8,11,14-Eicosatetraenoic acid, methyl
135	20.881	568401	2734-47-6	5,8,11,14,17-Eicosapentaenoic acid, meth
136	20.993	105349	14852-31-4	2-Hexadecanol
137	21.089	342492	1783-84-2	8,11,14-Eicosatrienoic acid, (Z,Z,Z)-
138	21.229	229346	7459-33-8	9,12-Octadecadienoyl chloride, (Z,Z)-

139	21.309	490008	629-96-9	1-Eicosanol
140	21.469	1.12E+06	19780-79-1	2-Hexyl-1-octanol
141	21.614	244282	130385-26-1	Z-28-Heptatriaconten-2-one
142	21.676	160683	55401-75-7	Anthracene, 9-dodecyltetradecahydro-
143	21.94	2.35E+06	1120-28-1	Eicosanoic acid, methyl ester
144	22.12	35613	765-14-0	Vinyl lauryl ether
145	22.187	88263	14852-31-4	2-Hexadecanol
146	22.269	81686	37577-58-5	Heneicosanoic acid, 2,3-dimethyl-, methy
147	22.411	130601	14852-31-4	2-Hexadecanol
148	22.524	443975	301-02-0	9-Octadecenamide, (Z)-
149	22.551	335277	14852-31-4	2-Hexadecanol
150	22.62	209570	301-02-0	9-Octadecenamide, (Z)-
151	22.7	66754	301-02-0	9-Octadecenamide, (Z)-
152	22.774	220715	14852-31-4	2-Hexadecanol
153	22.929	352599	None	2-Methyl-Z,Z-3,13-octadecadienol
154	23.015	57252	None	2-Methyl-Z,Z-3,13-octadecadienol
155	23.177	64269	3322-62-1	9-Octadecenamide
156	23.294	219744	119-47-1	Phenol, 2,2'-methylenebis[6-(1,1-dimethy
157	23.397	102776	None	Tricyclo[20.8.0.0(7,16)]triacontane, 1(2
158	23.528	2.70E+06	None	Tricyclo[20.8.0.0(7,16)]triacontane, 1(2
159	23.782	122206	56847-05-3	9-Octadecen-12-ynoic acid, methyl ester
160	23.867	49476	None	Oxalic acid, cyclohexylmethyl tridecyl e
161	24.006	777812	1472-93-1	Octadecanedioic acid, dimethyl ester
162	24.409	34297	127611-84-1	3',8,8'-Trimethoxy-3-piperidyl-2,2'-bina
163	24.506	87793	20548-62-3	Phthalic acid, bis(7-methyloctyl) ester
164	24.574	356226	661-19-8	1-Docosanol
165	24.662	574683	None	Oxalic acid, allyl octadecyl ester
166	24.741	108079	56630-69-4	13-Docosenoic acid, methyl ester
167	25.005	20957	14852-31-4	2-Hexadecanol
168	25.023	35488	130385-26-1	Z-28-Heptatriaconten-2-one

169	25.221	3.44E+06	117-81-7	Di-2-ethylhexyl phthalate
170	25.251	6.34E+06	117-81-7	Di-2-ethylhexyl phthalate
171	25.688	30231	127611-84-1	3',8,8'-Trimethoxy-3-piperidyl-2,2'-bina
172	25.805	29931	73105-67-6	1-Iodo-2-methylundecane
173	26.171	361660	None	Oxalic acid, allyl octadecyl ester
174	26.359	12229	55521-22-7	9,12,15-Octadecatrienoic acid, 2,3-bis[(
175	26.644	175727	2433-97-8	Tricosanoic acid, methyl ester
176	26.692	125711	2433-97-8	Tricosanoic acid, methyl ester
177	26.802	14197	None	Ethyl iso-allocholate
178	26.903	153738	54410-98-9	1-Nonene, 4,6,8-trimethyl-
179	27.065	123790	42235-38-1	Eicosanebioic acid, dimethyl ester
180	27.244	25598	55162-61-3	Tetracontane, 3,5,24-trimethyl-
181	27.285	18060	17453-58-6	3-Phorbinepropanoic acid, 9-acetyl-14-et
182	27.464	9556	None	.alpha.-d-Xylopyranoside, methyl-2,3,4-t
183	27.503	15645	56701-08-7	Carda-4,20(22)-dienolide, 3-[(6-deoxy-3-
184	27.537	11737	5767-82-8	5.alpha.-Pregn-16-en-20-one, 3.beta.,12.
185	27.637	618696	2425-77-6	1-Decanol, 2-hexyl-
186	27.718	27924	None	Oxalic acid, allyl octadecyl ester
187	27.743	76217	2425-77-6	1-Decanol, 2-hexyl-
188	27.856	151280	None	3,9.beta.;14,15-Diepoxypregn-16-en-20-on
189	27.939	48150	None	3,9.beta.;14,15-Diepoxypregn-16-en-20-on
190	28.102	2.67E+06	2442-49-1	Tetracosanoic acid, methyl ester
191	28.285	84730	2442-49-1	Tetracosanoic acid, methyl ester
192	28.325	277150	2442-49-1	Tetracosanoic acid, methyl ester
193	28.443	34265	2442-49-1	Tetracosanoic acid, methyl ester
194	28.475	37225	2442-49-1	Tetracosanoic acid, methyl ester
195	28.503	45709	2442-49-1	Tetracosanoic acid, methyl ester
196	28.526	62689	2442-49-1	Tetracosanoic acid, methyl ester
197	28.581	75510	17453-58-6	3-Phorbinepropanoic acid, 9-acetyl-14-et
198	28.624	36375	17453-58-6	3-Phorbinepropanoic acid, 9-acetyl-14-et

199	28.669	82163	2490-48-4	1-Hexadecanol, 2-methyl-
200	28.696	79656	17453-58-6	3-Phorbinepropanoic acid, 9-acetyl-14-et
201	28.757	24399	55521-22-7	9,12,15-Octadecatrienoic acid, 2,3-bis[(
202	28.784	44460	16695-32-2	2H-Pyran, 2-(7-dodecynyloxy)tetrahydro-
203	28.817	49848	150-86-7	Phytol
204	28.857	71777	17453-58-6	3-Phorbinepropanoic acid, 9-acetyl-14-et
205	29.045	713925	2425-77-6	1-Decanol, 2-hexyl-
206	29.239	940738	111-02-4	2,6,10,14,18,22-Tetracosahexaene, 2,6,10
207	29.367	56463	None	Tricyclo[20.8.0.0(7,16)]triacontane, 1(2
208	29.493	389348	55373-89-2	Pentacosanoic acid, methyl ester
209	29.556	166141	55373-89-2	Pentacosanoic acid, methyl ester
210	29.627	51538	7199-92-0	Cholesta-8,24-dien-3-ol, 4-methyl-, (3.b
211	29.647	28594	25328-53-4	Cholestan-3-one, cyclic 1,2-ethanediyl a
212	29.672	34385	17453-58-6	3-Phorbinepropanoic acid, 9-acetyl-14-et
213	29.712	35872	105-92-0	Rhodopin
214	29.744	37627	105-92-0	Rhodopin
215	29.783	19814	80097-22-9	17.beta.-Acetoxy-1',1'-dicarboethoxy-1.b
216	29.811	19162	7199-92-0	Cholesta-8,24-dien-3-ol, 4-methyl-, (3.b
217	29.921	298966	None	Ethyl iso-allocholate
218	29.988	54134	56599-45-2	9-Octadecenoic acid, (2-phenyl-1,3-dioxo
219	30.009	113060	30365-65-2	Ergost-8(14)-en-3-ol, (3.beta.)-
220	30.069	40185	17453-58-6	3-Phorbinepropanoic acid, 9-acetyl-14-et
221	30.089	32149	17453-58-6	3-Phorbinepropanoic acid, 9-acetyl-14-et
222	30.124	80507	17453-58-6	3-Phorbinepropanoic acid, 9-acetyl-14-et
223	30.301	10298	None	.pi.-Pentamethylcyclopentadienyl-trichlo
224	30.427	345932	195194-80-0	2-Piperidinone, N-[4-bromo-n-butyl]-
225	30.523	50641	55282-12-7	Octadecane, 3-ethyl-5-(2-ethylbutyl)-
226	30.573	42745	18835-33-1	1-Hexacosene
227	30.754	1586	75857-77-1	1'-Carboethoxy-1'-cyano-1.beta.,2.beta.-
Area Total:	1.28E+08			

PLFA determination of microbial community composition （TCP）

Sample Report

Sample ID: 3

Operator: zxy

Instrument ID: Varian GC/MS #1

Acquisition Date: 4/7/2016 20:21 PM

Method: D:\2015data\lina20151029\20140712.mth

Supplementary Table S10. PLFA determination of microbial community composition （TCP）

eaks: 265("Peak Numbe	RT (min)	Area	CAS Number	Name
1	5.258	2.85E+06	151-10-0	Benzene, 1,3-dimethoxy-
2	5.364	18580	17721-95-8	Piperidine, 2,6-dimethyl-1-nitroso-
3	5.43	1.40E+06	None	Oxalic acid, isobutyl nonyl ester
4	5.512	67964	55299-24-6	2H-1,4-Benzodiazepin-2-one, 7-chloro-1,3
5	5.578	41661	72439-85-1	3,5-Dibutoxy-1,1,1,7,7,7-hexamethyl-3,5-
6	5.699	4.30E+04	622-86-6	Benzene, (2-chloroethoxy)-
7	5.768	16101	877-44-1	Benzene, 1,2,4-triethyl-
8	5.812	52894	3913/2/8	1-Octanol, 2-butyl-
9	5.888	34349	13641-74-2	Dimethoxyamphetamine, 2,5-
10	5.951	28515	None	Oxalic acid, isobutyl nonyl ester
11	5.973	39172	29812-79-1	Hydroxylamine, O-decyl-
12	6.049	11920	540-97-6	Cyclohexasiloxane, dodecamethyl-
13	6.165	1.06E+06	None	Oxalic acid, isobutyl nonyl ester
14	6.358	282420	90-12-0	Naphthalene, 1-methyl-
15	6.503	191360	91-57-6	2-Methylnaphthalene
16	6.581	50506	None	Oxalic acid, dodecyl hexyl ester
17	6.626	21092	72439-85-1	3,5-Dibutoxy-1,1,1,7,7,7-hexamethyl-3,5-
18	6.683	49664	55134-08-2	Benzene, (2,3-dimethyldecyl)-
19	6.745	108237	29812-79-1	Hydroxylamine, O-decyl-
20	6.922	6.85E+05	5345-42-6	Benzene, 1-methoxy-3-methyl-2-nitro-

21	7.011	1.25E+06	None	N-HEXACOSANE
22	7.065	132221	131147-55-2	5-Ethenyl-5-(1-methyl-3-butenyl)-hexahyd
23	7.144	103005	30889-32-8	1,3-Dioxolane-4-methanol, 2-pentadecyl-,
24	7.23	138149	1127-76-0	Naphthalene, 1-ethyl-
25	7.357	476176	575-41-7	Naphthalene, 1,3-dimethyl-
26	7.434	44980	None	Alloaromadendrene oxide-(1)
27	7.5	550282	575-41-7	Naphthalene, 1,3-dimethyl-
28	7.553	342176	581-40-8	Naphthalene, 2,3-dimethyl-
29	7.691	201657	22198-47-6	4'-Ethoxy-2'-hydroxyoctanophenone
30	7.743	229090	575-41-7	Naphthalene, 1,3-dimethyl-
31	7.826	431406	719-22-2	2,5-Cyclohexadiene-1,4-dione, 2,6-bis(1,
32	7.922	270168	939-27-5	Naphthalene, 2-ethyl-
33	8.014	45773	55320-02-0	9,12,15-Octadecatrienoic acid, 2,3-bis(a
34	8.102	116453	None	N-HEXACOSANE
35	8.222	199040	644-08-6	1,1'-Biphenyl, 4-methyl-
36	8.265	1.47E+06	5875-45-6	Phenol, 2,5-bis(1,1-dimethylethyl)-
37	8.352	98934	643-58-3	1,1'-Biphenyl, 2-methyl-
38	8.403	156931	111-82-0	Dodecanoic acid, methyl ester
39	8.5	94606	None	3-(2-Methyl-propenyl)-1H-indene
40	8.523	105544	None	3-(2-Methyl-propenyl)-1H-indene
41	8.703	214880	620-47-3	Benzene, 1-methyl-3-(phenylmethyl)-
42	8.757	1.97E+05	829-26-5	Naphthalene, 2,3,6-trimethyl-
43	8.85	132621	2245-38-7	Naphthalene, 1,6,7-trimethyl-
44	8.926	152901	561-83-1	Barbituric acid, 5-allyl-5-neopentyl-
45	9.042	174159	14905-56-7	Tetradecane, 2,6,10-trimethyl-
46	9.127	68943	829-26-5	Naphthalene, 2,3,6-trimethyl-
47	9.274	157850	None	3-(2-Methyl-propenyl)-1H-indene
48	9.355	1.82E+05	31035-07-1	9-Nonadecene
49	9.459	1.02E+06	None	N-HEXACOSANE
50	9.602	148710	13151-91-2	Tridecane, 6-cyclohexyl-

51	9.669	49111	556-68-3	Cyclooctasiloxane, hexadecamethyl-
52	9.708	68452	143-28-2	Oleyl Alcohol
53	9.784	9888	7383-90-6	1,1'-Biphenyl, 3,4'-dimethyl-
54	10.048	93417	None	Epicedrol
55	10.148	86175	629-62-9	Pentadecane
56	10.231	30240	21895-14-7	Benzene, 1,1'-methylenebis[3-methyl-
57	10.413	42597	1454-84-8	1-Nonadecanol
58	10.546	35781	55282-12-7	Octadecane, 3-ethyl-5-(2-ethylbutyl)-
59	10.591	58832	None	Phthalic acid, methyl nonyl ester
60	10.773	153901	732-26-3	Phenol, 2,4,6-tris(1,1-dimethylethyl)-
61	10.814	162352	5129-58-8	Tridecanoic acid, 12-methyl-, methyl est
62	10.882	4.52E+04	61141-66-0	1,1'-Biphenyl, 3,4-diethyl-
63	11.014	407307	None	N-HEXACOSANE
64	11.227	94406	None	Methyl Z-11-tetradecenoate
65	11.325	5.91E+04	None	E-10-Pentadecenol
66	11.353	3.66E+04	None	2-Amino-6,7-dimethyl-5,6,7,8-tetrahydro-
67	11.426	527055	5129-58-8	Tridecanoic acid, 12-methyl-, methyl est
68	11.479	2.74E+04	27458-92-0	Isotridecanol-
69	11.559	18746	25117-30-0	4-Methyldocosane
70	11.719	127360	29812-79-1	Hydroxylamine, O-decyl-
71	11.854	57314	29812-79-1	Hydroxylamine, O-decyl-
72	11.959	50021	14852-31-4	2-Hexadecanol
73	12.049	71448	14852-31-4	2-Hexadecanol
74	12.093	84677	14852-31-4	2-Hexadecanol
75	12.21	288898	14852-31-4	2-Hexadecanol
76	12.318	5.69E+04	2825-81-2	2-Hexadecenoic acid, methyl ester, (E)-
77	12.51	2.22E+06	7132-64-1	Pentadecanoic acid, methyl ester
78	12.652	955536	5129-66-8	Tetradecanoic acid, 12-methyl-, methyl e
79	12.718	741276	None	N-HEXACOSANE
80	12.816	1.46E+05	None	Oxalic acid, allyl tridecyl ester

81	12.874	68093	2777-58-4	6-Octadecenoic acid, methyl ester, (Z)-
82	12.94	7.05E+04	36653-82-4	1-Hexadecanol
83	13.065	67135	2765/11/9	Pentadecanal-
84	13.159	486604	7132-64-1	Pentadecanoic acid, methyl ester
85	13.23	55587	629-83-4	Triacontanoic acid, methyl ester
86	13.268	81628	55682-91-2	Heptacosanoic acid, methyl ester
87	13.356	1.69E+05	102608-53-7	3,7,11,15-Tetramethyl-2-hexadecen-1-ol
88	13.454	130253	502-69-2	2-Pentadecanone, 6,10,14-trimethyl-
89	13.863	253320	84-78-6	1,2-Benzenedicarboxylic acid, butyl octy
90	13.918	204179	1120-25-8	9-Hexadecenoic acid, methyl ester, (Z)-
91	13.98	87766	5129-66-8	Tetradecanoic acid, 12-methyl-, methyl e
92	14.182	87761	36653-82-4	1-Hexadecanol
93	14.286	2.17E+06	5129-60-2	Pentadecanoic acid, 14-methyl-, methyl e
94	14.492	579018	22393-83-5	9-Hexadecenoic acid, hexadecyl ester, (Z
95	14.581	1.24E+06	1120-25-8	9-Hexadecenoic acid, methyl ester, (Z)-
96	14.752	1.36E+06	1120-25-8	9-Hexadecenoic acid, methyl ester, (Z)-
97	14.967	6.39E+06	5129-60-2	Pentadecanoic acid, 14-methyl-, methyl e
98	15.096	8.21E+04	6386-38-5	Benzenepropanoic acid, 3,5-bis(1,1-dimet
99	15.184	4.17E+04	None	Oxalic acid, allyl undecyl ester
100	15.248	8.64E+04	2490-48-4	1-Hexadecanol, 2-methyl-
101	15.39	133332	74630-39-0	1-Undecene, 4-methyl-
102	15.492	4.02E+04	None	Oxalic acid, allyl dodecyl ester
103	15.593	363306	10152-69-9	Cyclopropanenonanoic acid, 2-[(2-butylcy
104	15.647	627140	56875-67-3	7-Hexadecenoic acid, methyl ester, (Z)-
105	15.716	1.99E+06	2490-49-5	Hexadecanoic acid, 14-methyl-, methyl es
106	15.786	337776	55044-54-7	11-Hexadecenoic acid, 15-methyl-, methyl
107	15.847	741109	2490-49-5	Hexadecanoic acid, 14-methyl-, methyl es
108	15.911	154040	2825-81-2	2-Hexadecenoic acid, methyl ester, (E)-
109	16.087	1.55E+06	2490-49-5	Hexadecanoic acid, 14-methyl-, methyl es
110	16.172	87952	112-80-1	Oleic Acid

111	16.24	986819	2490-49-5	Hexadecanoic acid, 14-methyl-, methyl es
112	16.274	549128	None	N-HEXACOSANE
113	16.33	345494	56875-67-3	7-Hexadecenoic acid, methyl ester, (Z)-
114	16.48	5.08E+05	10152-61-1	Cyclopropaneoctanoic acid, 2-hexyl-, met
115	16.606	121724	112-80-1	Oleic Acid
116	16.693	48577	5353-25-3	Ethanol, 2-(9-octadecenyloxy)-, (Z)-
117	16.792	2.11E+06	2490-25-7	Heptadecanoic acid, 10-methyl-, methyl e
118	16.892	89006	54934-57-5	Heptadecanoic acid, 9-methyl-, methyl es
119	17.036	2.25E+05	55520-89-3	Hexadecanoic acid, trimethylsilyl ester
120	17.25	1.87E+05	18772-36-6	Cyclodecasiloxane, eicosamethyl-
121	17.461	808355	2490-25-7	Heptadecanoic acid, 10-methyl-, methyl e
122	17.555	236028	42199-20-2	Cyclopropanepentanoic acid, 2-undecyl-,
123	17.664	101762	59149-01-8	Methyl (Z)-5,11,14,17-eicosatetraenoate
124	17.757	304339	None	Oleanitrile
125	17.82	230412	1454-85-9	1-Heptadecanol
126	17.874	1.84E+05	112-61-8	Octadecanoic acid, methyl ester
127	17.961	4.05E+06	112-63-0	9,12-Octadecadienoic acid (Z,Z)-, methyl
128	18.095	1.14E+07	2716-53-2	2,3-Dihydroxypropyl elaidate
129	18.178	2.13E+06	112-62-9	9-Octadecenoic acid (Z)-, methyl ester
130	18.26	505298	112-62-9	9-Octadecenoic acid (Z)-, methyl ester
131	18.351	537881	112-62-9	9-Octadecenoic acid (Z)-, methyl ester
132	18.531	3.15E+06	112-61-8	Octadecanoic acid, methyl ester
133	18.606	351187	10152-62-2	Cyclopropaneoctanoic acid, 2-octyl-, met
134	18.705	75913	None	Z,E-3,13-Octadecadien-1-ol
135	18.781	69933	18835-33-1	1-Hexacosene
136	18.898	128386	40710-43-8	1-Pentacontanol
137	19.015	57217	871-70-5	Octadecanedioic acid
138	19.081	106523	None	Oxalic acid, allyl nonyl ester
139	19.201	1.21E+06	55124-97-5	Octadecanoic acid, 17-methyl-, methyl es
140	19.244	955814	55124-97-5	Octadecanoic acid, 17-methyl-, methyl es

141	19.38	96196	55124-97-5	Octadecanoic acid, 17-methyl-, methyl es
142	19.475	531116	629-54-9	Hexadecanamide
143	19.619	467057	556-71-8	Cyclononasiloxane, octadecamethyl-
144	19.685	8.79E+04	593-03-3	3-Hexadecanol
145	19.781	635791	630-06-8	Hexatriacontane
146	19.855	59477	14852-31-4	2-Hexadecanol
147	20.027	3.74E+06	10152-62-2	Cyclopropaneoctanoic acid, 2-octyl-, met
148	20.147	122179	629-96-9	1-Eicosanol
149	20.271	649487	1120-28-1	Eicosanoic acid, methyl ester
150	20.376	64108	None	Serverogenin acetate
151	20.458	68529	18748-91-9	Octadecanoic acid, trimethylsilyl ester
152	20.701	1.08E+06	505-54-4	Hexadecanedioic acid
153	20.759	475774	2566-89-4	5,8,11,14-Eicosatetraenoic acid, methyl
154	20.874	184442	2566-89-4	5,8,11,14-Eicosatetraenoic acid, methyl
155	20.989	3.55E+04	150-86-7	Phytol
156	21.083	1.07E+05	None	Z,E-2,13-Octadecadien-1-ol
157	21.177	53273	56630-73-0	9,15-Octadecadienoic acid, methyl ester
158	21.227	95148	None	3-(1,5-Dimethyl-hex-4-enyl)-2,2-dimethyl
159	21.306	200056	629-96-9	1-Eicosanol
160	21.461	734291	40710-43-8	1-Pentacontanol
161	21.599	195909	72845-33-1	1,6-Octadiene, 3-ethoxy-3,7-dimethyl-
162	21.677	81944	55334-01-5	Phenanthrene, 9-dodecyltetradecahydro-
163	21.746	73111	14852-31-4	2-Hexadecanol
164	21.932	1.69E+06	1120-28-1	Eicosanoic acid, methyl ester
165	22.013	124354	4130-54-5	Stearic acid hydrazide
166	22.078	53423	14852-31-4	2-Hexadecanol
167	22.176	70151	18835-33-1	1-Hexacosene
168	22.262	95757	37577-58-5	Heneicosanoic acid, 2,3-dimethyl-, methy
169	22.419	196531	14852-31-4	2-Hexadecanol
170	22.538	4.93E+06	301-02-0	9-Octadecenamide, (Z)-

171	22.754	616073	301-02-0	9-Octadecenamide, (Z)-
172	22.848	172929	301-02-0	9-Octadecenamide, (Z)-
173	22.93	772930	301-02-0	9-Octadecenamide, (Z)-
174	23.166	126947	301-02-0	9-Octadecenamide, (Z)-
175	23.29	554800	119-47-1	Phenol, 2,2'-methylenebis[6-(1,1-dimethy
176	23.39	1.05E+05	None	Tricyclo[20.8.0.0(7,16)]triacontane, 1(2
177	23.406	130847	None	Tricyclo[20.8.0.0(7,16)]triacontane, 1(2
178	23.522	921487	None	Tricyclo[20.8.0.0(7,16)]triacontane, 1(2
179	23.585	118764	None	2-Methyl-Z,Z-3,13-octadecadienol
180	23.604	461159	None	2-Methyl-Z,Z-3,13-octadecadienol
181	23.778	150511	None	2-Myristynoyl pantetheine
182	23.848	42841	None	Oxalic acid, butyl cyclohexylmethyl este
183	23.866	64390	18835-33-1	1-Hexacosene
184	24.206	123305	30889-32-8	1,3-Dioxolane-4-methanol, 2-pentadecyl-,
185	24.227	33804	30889-32-8	1,3-Dioxolane-4-methanol, 2-pentadecyl-,
186	24.247	42239	55320-07-5	Heptanoic acid, docosyl ester
187	24.309	68039	18835-33-1	1-Hexacosene
188	24.328	63862	17453-58-6	3-Phorbinepropanoic acid, 9-acetyl-14-et
189	24.45	177464	None	Phthalic acid, 2-ethylhexyl isohexyl est
190	24.578	1.02E+06	629-96-9	1-Eicosanol
191	24.654	676015	2425-77-6	1-Decanol, 2-hexyl-
192	24.745	263298	18835-33-1	1-Hexacosene
193	24.819	81754	18835-33-1	1-Hexacosene
194	24.896	58979	18835-33-1	1-Hexacosene
195	24.935	57177	74630-16-3	Phosphonous dichloride, (1,7,7-trimethyl
196	25.007	123569	14852-31-4	2-Hexadecanol
197	25.213	7.27E+06	117-81-7	Di-2-ethylhexyl phthalate
199	25.272	3.95E+06	117-81-7	Di-2-ethylhexyl phthalate
200	25.439	161055	4376-20-9	1,2-Benzenedicarboxylic acid, mono(2-eth
Area Total:		1.10E+08		
